# RNAe: an effective method for targeted protein translation enhancement by artificial non-coding RNA with SINEB2 repeat

**DOI:** 10.1093/nar/gkv125

**Published:** 2015-02-26

**Authors:** Yi Yao, Shouhong Jin, Haizhou Long, Yingting Yu, Zhenming Zhang, Ge Cheng, Chengwei Xu, Yan Ding, Qian Guan, Ning Li, Suneng Fu, Xiang-Jun Chen, Yong-Bin Yan, Hanshuo Zhang, Pei Tong, Yue Tan, Yang Yu, Shushu Fu, Juan Li, Guang-Jun He, Qiong Wu

**Affiliations:** 1 MOE Key Laboratory of Bioinformatics, School of Life Sciences, Tsinghua University, Beijing 100084, China; 2 State Key Laboratory of Biomembrane and Membrane Biotechnology, School of Life Sciences, Tsinghua University, Beijing 100084, China; 3 GenoArray Biotech, Suzhou 215123, China; 4 ViewSolid Biotech, Beijing 100085, China

## Abstract

In this study, a universal protein expression enhancement RNA tool, termed RNAe, was developed by modifying a recently discovered natural long non-coding RNA. At the moment, RNAe is the only technology for gene expression enhancement, as opposed to silencing, at the post-transcriptional level. With this technology, an expression enhancement of 50–1000% is achievable, with more than 200% enhancement achieved in most cases. This work identified the sufficient and necessary element for RNAe function, which was found to be merely 300 nucleotides long and was named minRNAe. It contains a 72-nt 5' pairing sequence which determines the specificity, a 167-nt short non-pairing interspersed nuclear element (SINE) B2 sequence which enhances ribosome recruitment to the target mRNA, and a poly(A) tail, provided together on a plasmid bearing the appropriate sequences. Cellular delivery of RNAe was achieved using routine transfection. The RNAe platform was validated in several widely-used mammalian cell lines. It was proven to be efficient and flexible in specifically enhancing the expression of various endogenous and exogenous proteins of diverse functions in a dose-dependent manner. Compared to the expression-inhibitory tool RNAi, the RNAe tool has a comparable effect size, with an enhancing as opposed to inhibitory effect. One may predict that this brand new technology for enhancing the production of proteins will find wide applications in both research and biopharmaceutical production.

## INTRODUCTION

Non-coding RNA transcripts are abundantly present in cells, but their functions are largely unknown. Some have been found to regulate gene expression at the transcriptional level. Carrieri*et al*. recently reported a new functional class of long non-coding RNAs (lncRNAs) with an overlapping antisense sequence targeting the 5' terminus of mRNAs, which enhances the translation of the corresponding protein at the post-transcriptional level, as in the case of ubiquitin carboxy-terminal hydrolase L1 (UCHL1) (1). This is most likely due to pairing of the lncRNA with the corresponding mRNA and enhanced ribosome recruitment. This recent discovery has expanded our views of expression regulation by non-coding RNAs. A bioinformatics study by the same group revealed more than 10, 000 lncRNAs with SINEB2 regions in the mouse genome (2). Among these, 31 sense-antisense RNA pairs were found where the antisense RNA transcript with embedded SINEB2-like element enhances the translation of the sense mRNA without increasing the mRNA level (1). A similar functional sense-antisense RNA pair was also found in rice, although the precise element in this antisense RNA which is responsible for translation enhancement remains unclear (3).

It was proposed that such antisense lncRNAs could be engineered to increase the translation efficiency of selected mRNAs. Here we report our efforts in this direction. The prototype antisense uchl1 lncRNA as reported by Carrieri*et al*. contains two essential parts—a 5' pairing segment and an inverted SINEB2 element. The 72 nucleotide (nt) pairing segment is complementary to the corresponding sense mRNA sequence around the translation initiation codon AUG and the head-to-head pairing of the sense and antisense RNA transcripts apparently results in efficient ribosome recruitment and enhanced translation. The inverted SINEB2 element is essential for translation enhancement but the exact mechanism has not been revealed yet.

Various natural antisense RNAs have been found to regulate the expression of the corresponding sense gene transcripts on different levels such as transcription (4,5), mRNA stability (6,7) and translation (8). However, except for microRNA (miRNA) and small interfering RNA (siRNA), no other RNA-based mechanism has been adapted as a wide-spread tool for expression regulation.

**Figure 1.**
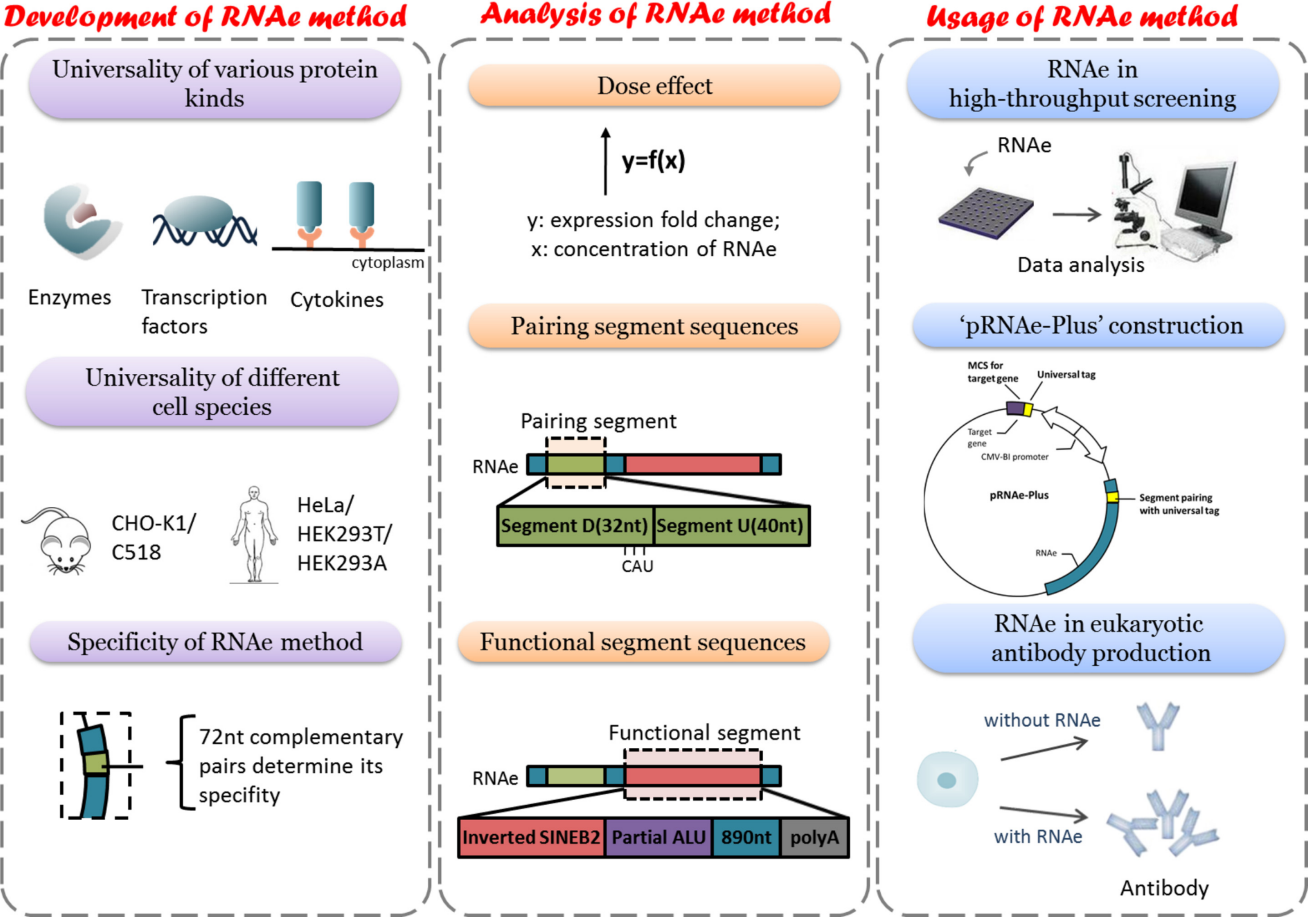
Graphical Abstract. RNAe was studied in three parts: development of the RNAe method, study and optimization of RNAe lncRNA elements, and finally the application of RNAe.

In the present study, we aim to demonstrate that artificial RNAs with an antisense sequence and an inverted SINEB2 element can be used as a universal molecular tool for translation enhancement of mammalian protein-coding genes. We named it RNAe, which is short for RNA enhancement. Much effort has been made in this study to broaden the scope of RNAe to make it universally applicable, as well as in optimization to achieve higher efficiency under various conditions (Figure 1).

## MATERIALS AND METHODS

### Cell culture

HeLa, HEK293A, HEK293T and C5.18 cells were cultured in Dulbecco's modified Eagle medium (DMEM) (Gibco, USA) containing 10% fetal bovine serum (FBS) (Gibco, USA), 100 units/ml penicillin (Biodee, China) and 0.1 mg/ml streptomycin (Biodee, China). Rat primary mesenchymal stem cells (MSCs) were cultured in MSC medium (MSCM) (Sciencell, USA). Rat primary chondrocytes were cultured in DMEM containing 10% FBS, 100 units/ml penicillin, 0.1 mg/ml streptomycin and 25 mg/L L-ascorbic acid-2 phosphate (Sigma Dorset, UK). CHO-K1 cells were cultured in F12 medium (Gibco, USA) containing 10% FBS, 100 units/ml penicillin and 0.1 mg/ml streptomycin. All cells were cultured in humidified conditions with 95% air and 5% CO_2_ at 37°C.

### Cell transfection

Transfection experiments were conducted using the Lipofectamine 3000 (Invitrogen, USA) transfection reagent, according to the manufacturer's protocol. Plasmids for transfection were constructed as shown in Supplementary Table S3.

Unless the cell number and well size information are stated specifically, in each transfection experiment, cells were passaged on 12-well plates. When cell confluence reached 40–50%, plasmid transfection was conducted using Lipofectamine 3000 reagent. Unless stated explicitly, the plasmid concentrations used for transfection were as follows: For pRNAe and plasmid expressing target gene co-transfection, 0.2 μg expression plasmid and 1.2 μg of pRNAe plasmid or control plasmid were used per well of 12-well plates; for the expression of the light-chain and heavy-chain of anti-HIV antibody 10E8, 0.2 μg of each and 1.2 μg of pRNAe plasmid or control plasmid were used per well of 12-well plates. For transfection experiments with only one plasmid, we used 1 μg plasmid per well of 12-well plates. For pRNAe-Plus experiments, the amount of pRNAe-Plus plasmid was as listed in the figure, while the total DNA amount per well was kept constant by supplementing to 1.5 μg with an appropriate amount of pRNAe-Mock plasmid. Cells were analyzed 2 days after transfection, unless otherwise specified.

RNA without 5' cap for transfection was transcribed *in vitro* by HiScribe™ T7 High Yield RNA Synthesis Kit (New England Biolabs, UK). RNA with 5' cap was transcribed also by HiScribe™ T7 High Yield RNA Synthesis Kit with m7G(5′)ppp(5′)G (New England Biolabs, UK) added. The concentration for transfection was 0.15 μg per well of 96-well plates for the subsequent CCK8 assay.

### Flow-cytometry (FCM) measurement

For FCM-based fluorescence measurements, cells from each group were trypsinized, and re-suspended in phosphate buffered saline (PBS) containing 0.5% FBS. Green fluorescent protein (GFP) fluorescence was measured using a BD Flow Cytometry Calibur (BD Biosciences, USA) using the 488nm laser.

### Quantitative real-time polymerase chain reaction (qRT-PCR)

Total RNA was isolated from treated cells using the miRspin mRNA Isolation Kit (Tiangen, China). Subsequently, cDNA for mRNA analysis was synthesized using Fastquant RT Kit (Tiangen, China). qRT-PCR was performed using SuperReal PreMix (Taqman probe) (Tiangen, China). Glyceraldehyde-3-phosphate dehydrogenase (GAPDH) mRNA was used as internal control for mRNA detection. The primers and probes used for qRT-PCR are listed in Supplementary Table S4.

### Western blot analysis

Whole-cell lysates for western blot were extracted with RIPA Lysis Buffer (Beyotime, China). Anti-SOX9, -GFP and -BBCK antibodies were obtained from Millipore (Billerica, USA), HX-BIO (China) and Santa Cruz (USA), respectively. The secondary antibodies used for western blot detection, conjugated with horseradish peroxidase (HRP), were obtained from Sigma (Sigma-Aldrich, USA). The blots were developed using enhanced chemiluminescence (ECL) reagents (Pierce Biotechnology, USA) and visualized using a GE LAS4000 station (General Electric Company, USA).

### Polysome profiling

Polysome profiles were obtained using sucrose density gradient centrifugation. HEK293T cells transfected with the indicated plasmids were treated with 100 μg/ml cycloheximide (CHX) (Biodee, China) for 10 min prior to lysis in 150 μl of lysis buffer comprising 10 mM HEPES pH 7.4, 5 mM MgCl_2_, 150 mM KCl, 0.5% NP-40 (Biodee, China), 100 μg/ml cycloheximide, 1 mM dithiothreitol (DTT) (Biodee, China), 200 U each of RNase-in (Tiangen, China) and protease inhibitor (EDTA-free) (HX-bio, China). Whole-cell extracts were clarified by centrifugation at 13,000 g and 4°C for 10 min. 300 μl of the clarified cell extract was layered on top of a linear 20–45% (w/v) sucrose gradient in a buffer comprising 10 mM HEPES pH 7.4, 5 mM MgCl_2_, 150 mM KCl, 100 μg/ml cycloheximide and 1 mM DTT, and centrifuged at 112 000 g at 4°C for 2.5 h. The gradient was pumped out by upward displacement and the absorbance at 260 nm was monitored using a Piston Gradient Fractionator (BIOCOMP, Canada). Fractions were collected based on the 260 nm peak, and RNA was extracted by the addition of 1.5 ml of Trizol LS reagent (Invitrogen, USA), following the manufacturer's instructions.

### Alcian blue staining

The treated cells were fixed in 4% paraformaldehyde (Dingguo Changsheng Biotechnology Co. Ltd., China) for 5 min, and then incubated with 1% Alcian Blue (Cyagen, Guangdong, China) for 15 min for primary chondrocytes or 30 min for C5.18 cells. Excess stain was washed away with PBS. Images were captured by a light microscope for analysis.

### 3' rapid amplification of cDNA ends (RACE)

Total RNA extracts from HEK293T cells transfected with RNAe plasmids were treated with DNase I (Tiangen, China) and reverse transcription was done using the TaKaRa 3'-Full RACE Core Set with PrimeScript RTase (TaKaRa, Japan), according to the manufacturer's protocol. 3'RACE-primer-1 (Supplementary Table S4) was used for the first round of PCR amplification and 3'RACE-primer-2 (Supplementary Table S4) was used for the second round nested PCR amplification. PCR products were identified using agarose gel electrophoresis in gels containing 3% agarose, and extracted from the gels for sequencing.

### High-throughput CCK8 assay

Cells were trypsinized in the culture medium and cell density was measured immediately using a Scepter 2.0 Handheld Automated Cell Counter (Milipore, USA). Subsequently, cells were dispensed into 96-well culture plates (Costar, USA) using an automatic cell dispenser (Multidrop Combi, Thermo, USA), incubated for ∼12 h to recover and subsequently transfected with Lipofectamine 2000 (Invitrogen, USA). The plasmid or RNA concentration used for transfection was 0.15 μg per well of 96-well plates. The media was changed 4 h after transfection to normal fresh medium to remove the transfection reagent and the cells were subsequently incubated for 20 h (DMEM with 100 units/ml penicillin and 0.1 mg/ml streptomycin without FBS or other protein supplement provided for FBS starvation group) to induce apoptosis. As control, a fresh aliquot of the same medium with 10% FBS was used. After about 20 h of starvation, CCK8 reagent (Cell Counting Kit (CCK-8/WST-8), QC-Bio, China) was added and the wells were screened using an automatic microplate reader (Enspire, Multimode Reader, USA). The absorbance at 450 nm was recorded.

### Antibody sample preparation

HEK293T cells were passaged to 12-well plates. When cell confluence reached 70–90%, the indicated plasmids (control plasmid, antibody plasmid or corresponding RNAe plasmid) were transfected using a total amount of 1.6 μg plasmid DNA per well (0.2 μg each of plasmid DNA encoding light-chain, and heavy-chain of anti-HIV antibody 10E8 and 1.2 μg of either pRNAe plasmid or control plasmid), using Lipofectamine 3000 (Invitrogen, USA). Supernatants of so treated HEK293T cells were refreshed at 24 h after transfection and collected 24–72 h after transfection for ELISA, as described below.

### Enzyme-linked immunosorbent assay (ELISA)

Peptide antigen recognized by anti-HIV antibody 10E8 (0.2 μg/ml of peptide MPER RRRNEQELLELDKWASLWNWFDITNWLWYIRRRR, Chinese Peptide Company, China, 50 μl per well) was coated onto 96-well ELISA plates (Biodee, China) in 0.1 M NaHCO_3_ buffer (pH 9.6) at 4°C overnight. Subsequently, the plates were blocked with 0.3% gelatin (Biodee, China) (200 μl per well) at 37°C for 2 h. The collected cell culture supernatants (50 μl per well) were added and the reactions incubated at 37°C for 2 h. After washing three times with PBS-T (PBS pH 7.4 (Dingguo Changsheng Biotechnology Co. Ltd., China) containing 0.05% Tween 20 (Sionpharm Chemical Reagent, China)), the binding of antibody to antigen was detected with an HRP-conjugated goat anti-human antibody (HX-BIO, China) and TMB (3, 3, 5, 5 tetramethylbenzidine, Boster Biological Technology, China) using an automatic microplate reader (Enspire, Multimode Reader, USA). The absorbance at 450 nm was recorded.

### Quantitative mass spectrometry

Total protein of transfected cells was extracted the same way as for the western blot, and equal amounts of samples were separated on a 12% SDS-PAGE gel and TMT-based (tandem mass tags) quantitative mass spectrometry (QMS) was applied as published previously(9).

### Time-lapse fluorescence accumulation assay

HEK293T cells co-transfected with pEGFP-C1, and either pRNAe-mock or pRNAe-egfpc1 plasmid was cultured for 6 h after transfection. Living cell fluorescence images were taken using a Nikon Ti-E inverted microscope (Nikon, Japan) every 10 min for 19 h. Relative average fluorescence strength was calculated using the NIS-Element C software (Nikon, Japan).

### Statistical analysis

Statistical analysis was performed using the paired two-tailed Student's *t*-test. Results are given as mean (*n* ≥ 3) ± standard deviation (SD). Values were considered significant at *P* < 0.05, *P* < 0.01 or *P* < 0.001, and indicated with one, two or three stars, respectively.

## RESULTS

### Validation of RNAe-mediated enhancement of EGFP expression through ribosome recruitment

**Figure 2.**
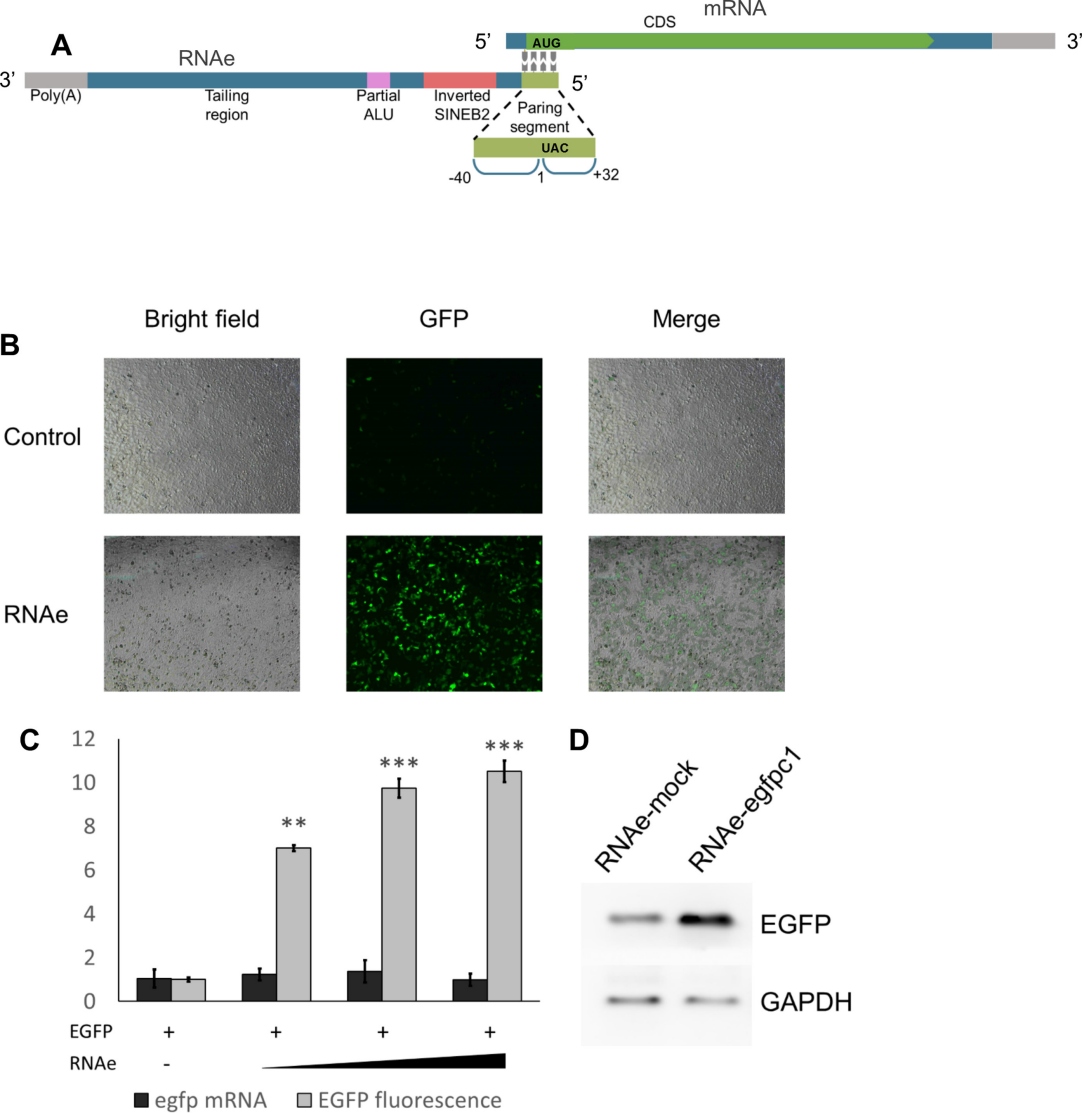
Development of RNAe. (**A**) Sketch map of RNAe lncRNA and its target mRNA. (**B**) Effect of RNAe measured by fluorescence spectroscopy in HEK293T cells co-transfected with 0.2 μg of pEGFP-C1 plasmid and 1.2 μg of pRNAe-Mock plasmid (Control group) or 1.2 μg of pRNAe-egfpc1 plasmid (RNAe group). (**C**) Measurement of relative mRNA levels and fluorescence of RNAe-mediated enhancement of EGFP expression (pEGFP-C1 plasmid amount in each group was 0.2 μg, and pRNAe plasmid amounts were 0, 0.6, 1.2 and 1.8 μg, respectively) (mean ± SD, *n* = 4. **P < 0.01, ***P < 0.001, two tailed *t*-test). (**D**) RNAe-mediated enhancement of EGFP expression visualized by western blot, same constructs as listed above.

**Figure 3.**
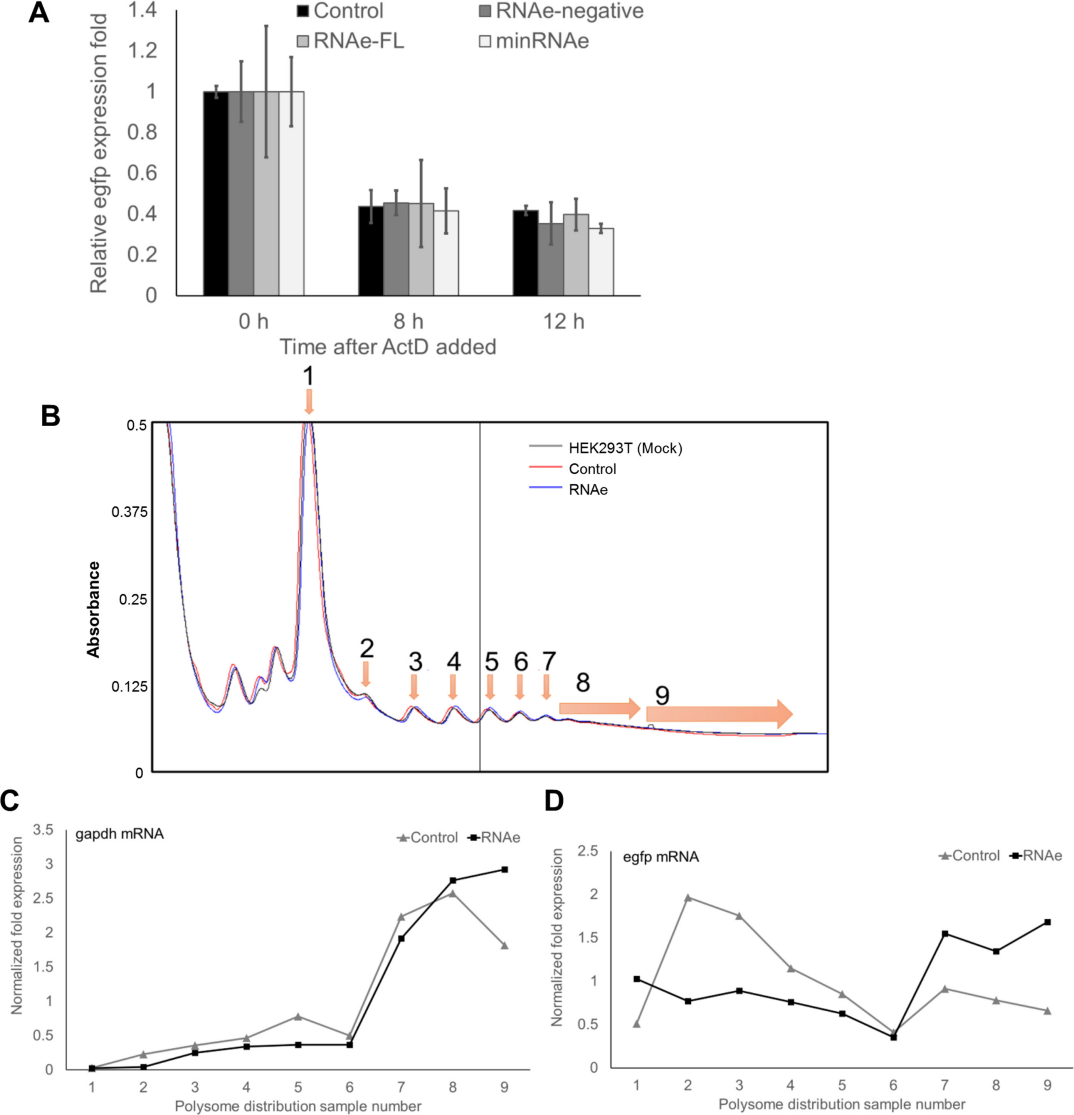
Predicted mechanism of RNAe. (**A**) Actinomycin D (ActD) was added to HEK293T cell culture medium for 0, 8 or 12h to impair mRNA generation after pEGFP and pRNAe were transfected. qRT-PCR results showed that RNAe did not impair corresponding mRNA stability (mean ± SD, *n* = 3). (**B**) The total polysome distribution with (RNAe, blue line)/without (Control, red line) RNAe lncRNA and normal HEK293T cells (Mock) without transfection (gray) to ensure the total polysome distribution of RNAe or control group is normal. (**C** and **D**) Polysome distribution of egfp mRNA in HEK293T cells (D), with gapdh as internal control (C), with RNAe transfection (RNAe group) or without transfection (Control group).

We first validated the finding of Carrieri*et al*., using an original construct kindly provided by the group. The plasmid pRNAe-egfpc1, carrying the antisense uchl1 lncRNA with a modified pairing sequence for egfp as in pEGFP-C1 and the functional sequence containing an inverted SINEB2 sequence (Figure 2A), was tested in co-transfection experiments. Co-transfection of pEGFP-C1 and pRNAe-egfpc1 (or pRNAe-mock plasmid) into HEK293T cells revealed that EGFP protein expression, as assessed by fluorescence flow-cytometry and Western blot analysis(Figure 2 B, C and D), was significantly higher in the RNAe group compared to the mock control group. On the other hand, egfp mRNA expression, as assessed by quantitative real-time polymerase chain reaction (qRT-PCR), showed little difference as is also shown in Figure 2C. This result demonstrates that RNAe-egfpc1 enhances EGFP protein expression at the post-transcriptional level, which was consistent with the observations shown by Carrieri*et al*. The qRT-PCR results from HEK293T cells transfected with RNAe showed that RNAe did not have an effect on the mRNA level of the target, EGFP. In a separate experiment, Actinomycin D (ActD) was used to block transcription, and qRT-PCR results showed that RNAe also did not influence the degradation rate of egfp mRNA (Figure 3A), also indicating that it exerts its effect at the post-transcriptional level. In order to quantify the enhancement effect of RNAe, a time-lapse fluorescence accumulation assay was conducted. HEK293T cells were transfected with different doses of RNAe plasmid and the fluorescence of the cells was measured in a time-lapse series every 10 min. The result showed that RNAe not only enhanced the total accumulation of fluorescence but also accelerated its expression (Supplementary Figure S1).

To further confirm that RNAe acts via recruiting polysomes to the target mRNA, as had been speculated by Carrieri*et al*., polysome profiling and qRT-PCR was carried out with the above-mentioned co-transfected HEK293T cells. Polysomes with the associated mRNAs were extracted from HEK293T cells contained in a 15 cm dish, co-transfected with 5 μg pEGFP-C1 and either 35 μg pRNAe-egfpc1 or 35 μg of pRNAe-mock, and were separated by sucrose gradient. The overall polysome distribution pattern was almost identical in the RNAe-egfpc1 and the RNAe-mock cells (Figure 3B). However, egfp mRNAs preferentially associated with heavier polysomes (*n* = 7 or higher) when cells were transfected with RNAe (Figure 3D), whereas the association pattern of glyceraldehyde phosphate dehydrogenase (gapdh, internal control) mRNAs did not differ (Figure 3C). This result confirmed what had been observed by Carrieri*et al*., and suggested that GFP-specific RNAe increased the number of ribosomes associated with the respective target mRNAs and thus enhanced their translation level.

### Wide applications of RNAe to specifically enhance protein expression

Next we tested whether RNAe could be universally applied to enhance protein expression in a specific manner. We wanted to answer the following questions: 1) What systems could it be applied to? 2) Can RNAe work with every protein? 3) What are the determinants of specificity?

**Figure 4.**
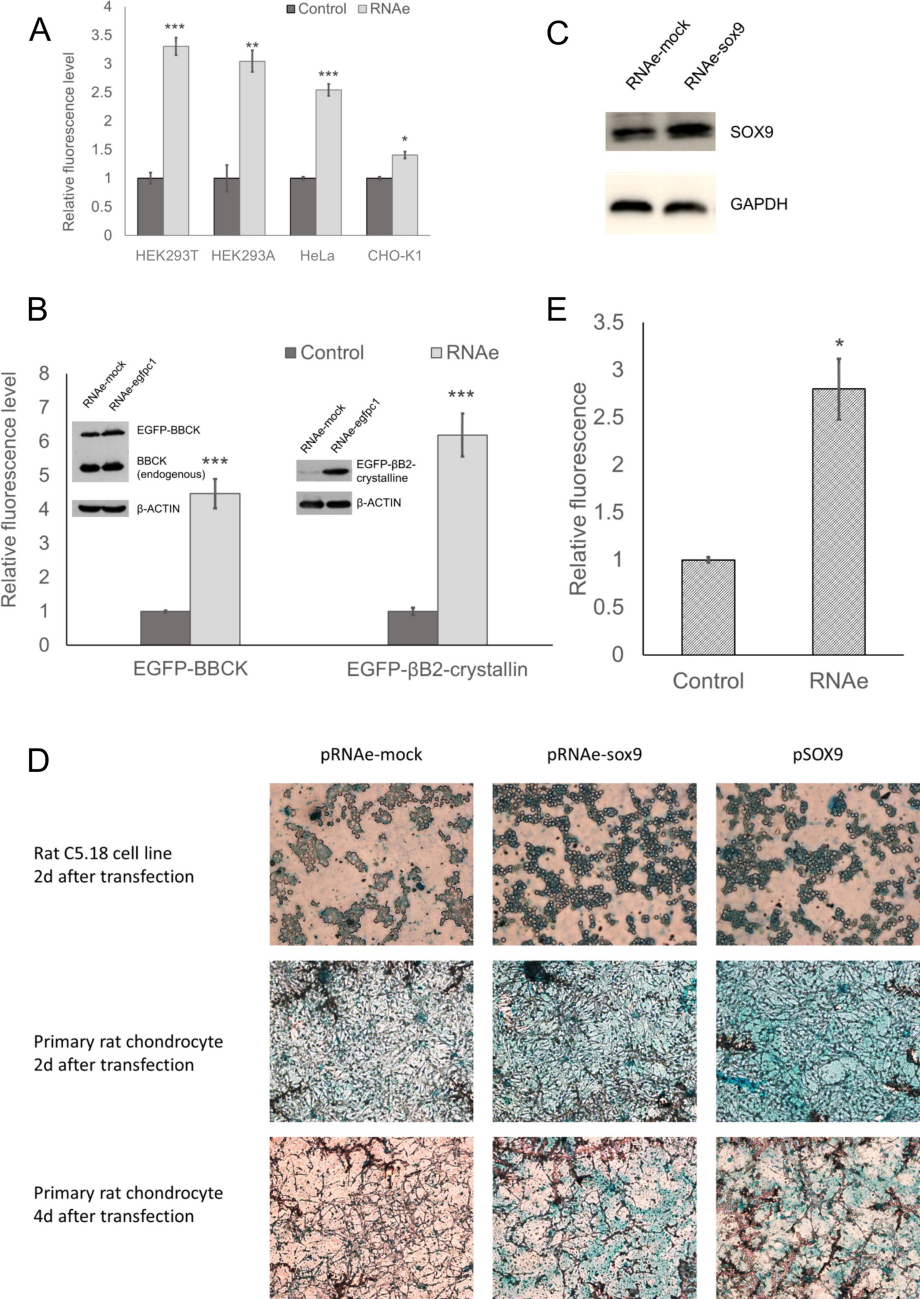
Wide applications of RNAe. (**A**) RNAe-mediated enhancement in various cell types (mean ± SD, *n* = 4. *P < 0.05, **P < 0.01, ***P < 0.001, two tailed *t*-test). (**B**) Relative fluorescent level and protein level of EGFP-BBCK and EGFP-βB2-crystalline under RNAe enhancement in HEK293T cells (mean ± SD, *n* = 4. ***P < 0.001, two tailed *t*-test). (**C** and **D**) Relative Sox9 expression level (C) and alcian blue staining (D) of sGAG in C5.18 cell and rat primary chondrocyte transfected with RNAe targeting endogenous sox9 mRNA. (**E**) Relative expression level of secretory MET-LUCIFERASE under RNAe enhancement (mean ± SD, *n* = 6. *P < 0.05, two tailed *t*-test).

We first tested the RNAe effect of pRNAe-egfpc1 on EGFP expression from pEGFP-C1 in several cell lines widely used in research and eukaryotic protein production, such as human cells lines HEK293T, HEK293A, HeLa, and Chinese hamster ovary cell line CHO-K1 (Figure 4A). Fluorescence analysis by flow-cytometry indicated that RNAe-egfpc1 significantly enhanced EGFP green fluorescence in all these cell lines, although the effect was not equally strong in all cases.

Next we demonstrated that in HEK293T cells the co-transfection of pRNAe-egfpc1 with pEGFP-BBCK (encoding a fusion protein comprising N-terminal EGFP and C-terminal brain-type creatine kinase) enhanced the transient expression of protein EGFP-BBCK as indicated by fluorescence and western blot analysis. Furthermore, the construct did not affect the expression of the endogenous BBCK, which had a high endogenous expression level in most cell lines, as indicated by western blot analysis (Figure 4B). This result suggests that the enhancement was specific for the pairing sequence complementary to the exogenous N-terminal EGFP sequence rather than to the endogenous BBCK mRNA sequence. Similarly, co-transfection of pRNAe-egfpc1 and pEGFP-βB2-crystallin (encoding a fusion protein comprising N-terminal EGFP and C-terminal βB2-crystallin, a lens-specific protein with little expression in most cell lines) into HEK293T cells enhanced EGFP-βB2-crystallin expression (Figure 4B). EGFP-βB2-crystallin was found difficult to express by regular methods, and was only visible in the western blot as a faint band in the absence of RNAe. These results demonstrated that RNAe can also be used to raise the expression level of a protein which is almost non-detectable under normal circumstances, to a level which is on par with the other model proteins studied.

### Moving beyond reporter proteins or fusion proteins, we tested whether RNAe could enhance expressions of endogenous proteins

SRY (sex determining region Y)-box 9 (SOX9) is a key transcription factor during chondrogenesis and can help the transcription of genes encoding downstream matrix proteins such as type II collagen (10). We constructed a pRNAe-sox9 plasmid with a pairing sequence complementary to rat endogenous sox9 mRNA and transfected the plasmid into rat pre-chondrocyte cell line C5.18 cells. Western blot analysis showed that pRNAe-sox9 transfection enhanced the expression level of endogenous SOX9 protein (Figure 4C) and the SOX9 protein overproduced using RNAe has been demonstrated to function normally using alcian blue staining to demonstrate chondrogenesis. This staining for chondrocyte specific matrix protein sulphated glycosaminoglycan (sGAG) of C5.18 cells showed that enhanced chondrogenesis was visible in RNAe-sox9, and not in the pRNAe-mock group, and was conparable to the sox9 cDNA overexpression (positive control) group (Figure 4D, top panel). The same set of experiments was carried out in rat primary chondrocytes of passage 2, where a satisfactory transfection efficiency of about 40–50% was achieved (11), and functional enhancement with the specific RNAe was also observed (Figure 4D, middle and bottom panel). Thus, this often used model rat cell line, as well as primary rat cells can be added to the list of cells where RNAe technology can be used.

In addition to cytosolic proteins, we demonstrated that the RNAe strategy can be applied to enhance the expression of secreted proteins, such as met-luciferase (a fusion protein of a secretory signal peptide and the reporter gene luciferase, Clontech, USA) (Figure 4E). We set this apart from cytosolic proteins because on one hand, translation of these proteins involves ribosomes bound to the endoplasmic reticulum rather than cytosolic “free” ribosomes; and on the other hand, secretion requires a signal peptide which might present with lower RNAe specificity since its encoding sequence might be found in a number of mRNAs. This possibility requires some consideration and exploration in RNAe design.

#### The specificity of RNAe to its target protein was further confirmed via proteomics analysis and cross-over experiments

HEK293T cells with stable EGFP expression were transfected with pRNAe-mock, pRNAe-negative (RNAe without pairing sequence) and pRNAe-egfpc1 plasmid DNA, respectively, and proteomics analysis was performed by quantitative mass spectrometry as described previously (9). No observable difference was found between these three groups (Supplementary Figure S2), indicating that RNAe does not affect cellular expression in general. Further cross-over experiments were designed to analyze the specificity of RNAe in a set of similar proteins. Three EGFP or EGFP fusion protein expression plasmids pEGFP-C1, pEGFP-N1, pPARN-EGFP were chosen for cross-over experiments to further test the specificity of RNAe. For pRNAe-egfpc1 (Supplementary Figure S3A and B), enhancement was observed with different kinds of EGFP constructs when combined with RNAe constructs complementary to either 5' UTR or 5' TR. However, the enhancement efficiency was significantly higher with the fully-overlapping target pEGFP-C1 compared to constructs with a shorter overlap. For pRNAe-egfpn1 (Supplementary Figure S3C and D) enhancement was seen in combination with a different EGFP construct with a complementary 5' TR (pEGFP-C1) but not in the case of pPARN-EGFP which has a shorter overlapping sequence. Similarly, the enhancement efficiency was significantly higher for the fully-overlapping target pEGFP-N1.

### Further study of RNAe lncRNA elements

The original antisense uchl1 RNA was transcribed from a long genomic sequence, and the RNA transcript was about 1.2 kilobases (kb) in length, including a 72 nt pairing sequence (−42 to +32), an inverted SINEB2 element, a partial ALU element, and a ∼890 nt tailing portion before the poly(A) tail (Figure 2A). Several experiments were designed to study the importance of each element individually, and based on the insight gained, RNAe cassettes were optimized for length of pairing region as well as total length.

#### The 5' TR of the pairing segment is essential for RNAe function and the pairing pattern critically affects RNAe function

**Figure 5.**
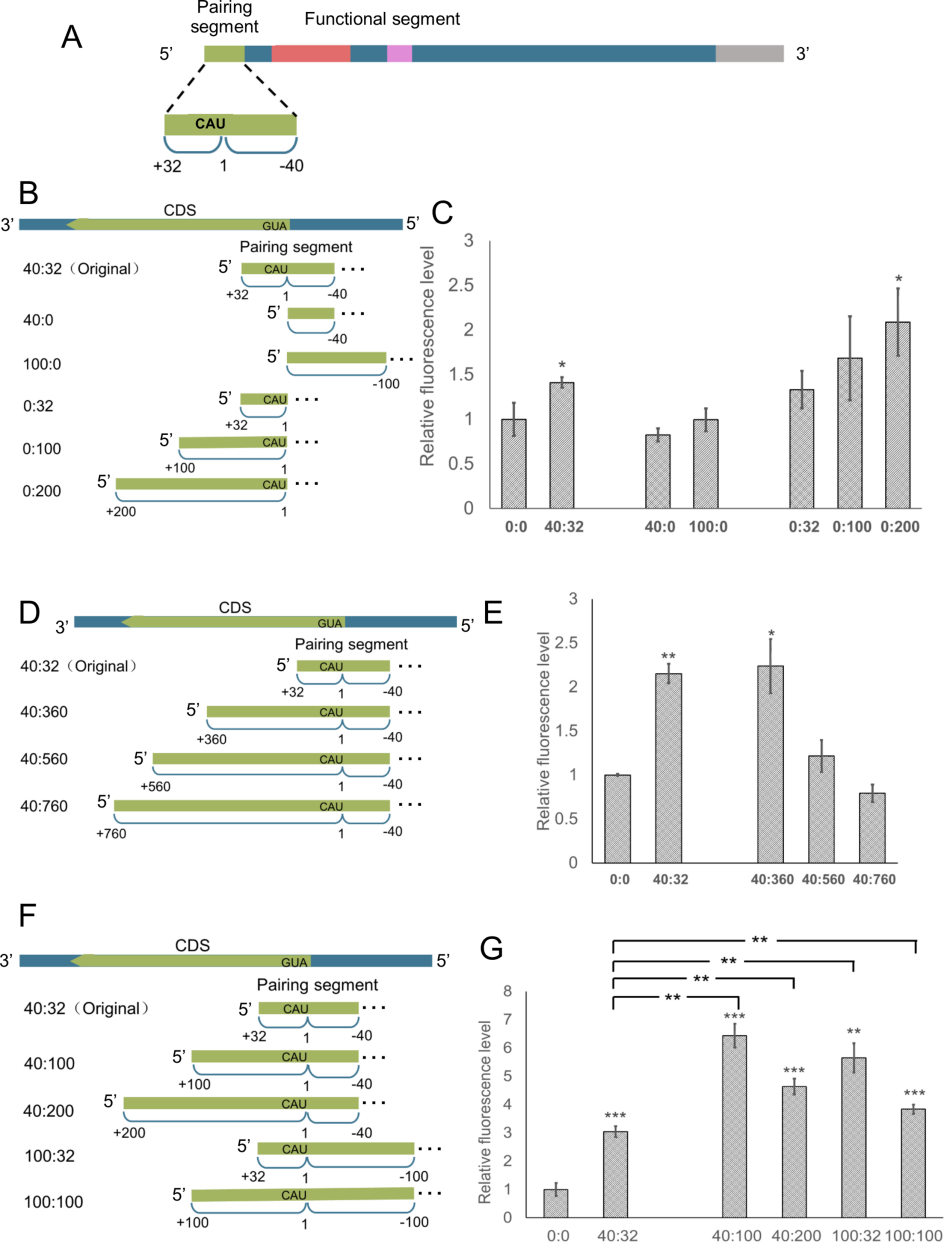
The pattern of the pairing segment of RNAe determines its enhancement efficiency of target protein expression. (**A**) Sketch map and detailed pairing pattern of original RNAe (the same pairing rule with AS Uchl1). (**B** and **C**) Importance of 5' TR or 5' UTR in pairing segments. Structure of pairing segments having only 5' TR or 5' UTR (B) and their effect on EGFP expression indicated by EGFP fluorescence in HEK293T cells (C). (**D** and **E**) Length effect of 5' TR on RNAe enhancement effect in HEK293T cells. Structure of extended 5' TR pairing segments (D) and their effect on EGFP expression indicated by EGFP fluorescence in HEK293T cells (E). (**F** and **G**) Optimization of the pairing segment of RNAe. Structure of optimized pairing segments (F) and their effect on EGFP expression indicated by EGFP fluorescence in HEK293T cells (G). The horizontal axis indicates numbers of nucleotides pairing to 5' UTR and 5' TR of target mRNA, respectively (mean ± SD, *n* = 4. *P < 0.05, **P < 0.01, two tailed *t*-test).

As the original RNAe pairs with the −40 nt to +32 nt region of the target mRNA (Figure 5A), we used the RNAe-egfpc1 with the same pairing segment length (expressed as 40:32) as the original control and a 0:0 pairing sequence (no pairing) as mock control. We further tested several RNAe constructs with pairing complementary only for the 5' untranslated region (5' UTR, 40:0 and 100:0), only the 5'-translated region (5' TR, 0:32, 0:100 and 0:200) (Figure 5B), or for the extended 5' TR (40:360, 40:560 and 40:760) (Figure 5D). These series of RNAe plasmids were co-transfected with pEGFP-C1 into HEK293T cells and EGFP green fluorescence was measured as already described. The results clearly show that the pairing sequence in the 5' TR is essential, as removing it (40:0) abolished the RNAe effect. Interestingly, prolonging the pairing in the 5' UTR (100:0) did not help; in contrast, a deletion of the pairing sequence in the 5' UTR had little influence on RNAe as long as the total length amounted to a threshold value such as 100 (0:100 or 0:200) (Figure 5C). Adding longer pairing sequences at 5' TR up to a certain length (total 400 nt) produced similar enhancement effect as the original 40:32. However, extending the pairing sequence up to 600 nt or 800 nt total produced only moderate or even inhibitory effects on target protein expression (Figure 5E). This might be due to the typical antisense pairing induced mRNA degradation mechanism. These results indicate that the original 40:32 pairing sequence is sufficient in terms of its length, region coverage and enhancement effect. We nevertheless do believe that optimization for a different protein may yield a better enhancement effect with a pairing distribution somewhat different from this standard 40:32 (Figure 5F and G). Since it was shown to be sufficient and robust, the 72 nt pairing sequence in 40:32 distribution was kept as the standard pairing sequence architecture for other RNAe experiments.

#### The SINEB2 sequence is sufficient for RNAe function

**Figure 6.**
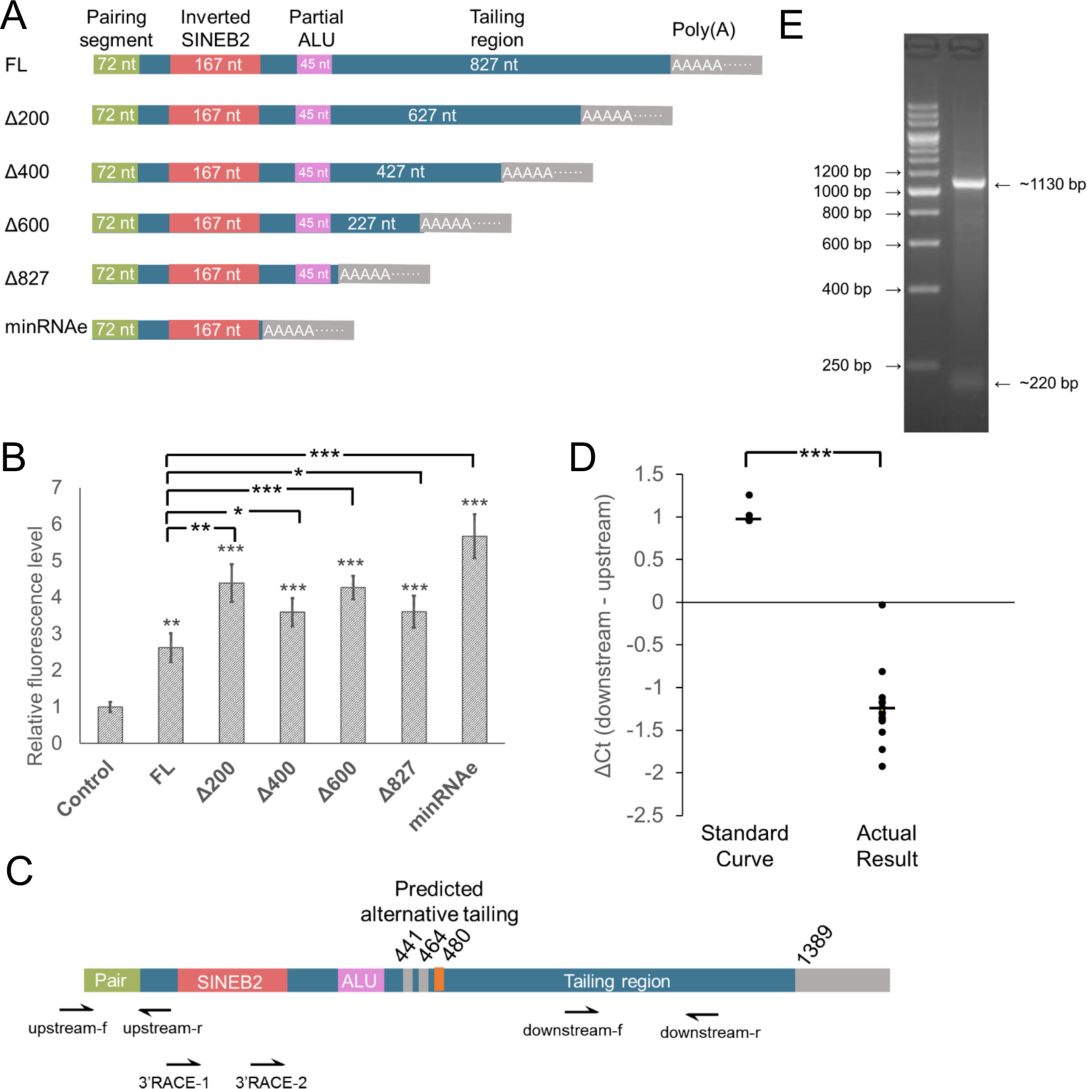
Truncations of RNAe confirmed that the SINEB2 element is sufficient for the RNAe effect. (**A** and **B**) Sketch map of the truncations (A) of RNAe-FL (full-length RNAe, the same sequence with AS Uchl1 except for the pairing element) and their effect on EGFP expression indicated by fluorescence in HEK293T cells (B) (mean ± SD, *n* = 4. *P < 0.05, **P < 0.01, ***P < 0.001, two tailed *t*-test). (**C**) Sketch map of the alternatively tailed lncRNA, including primers and probes used in qRT-PCR and 3'RACE. (**D** and **E**) Confirmation of the alternative tailing of the RNAe lncRNA by qRT-PCR (D) and 3'RACE (E) in HEK293T cells (standard curve, *n* = 5; actual result, *n* = 15. ***P < 0.001, two tailed *t*-test).

As original RNAe had a long functional region sequence (the sequence after pairing sequence which may provide RNAe function), we made a series of truncated constructs of RNAe-egfpc1 as shown in Figure 6A. The constructs were co-transfected with pEGFP-C1 into HEK293T cells, and EGFP fluorescence data showed that the highest enhancement was seen with the shortest RNAe construct (Figure 6B), the 282 nt construct termed minRNAe. This construct contained only the 72 nt pairing sequence, an inverted SINEB2 sequence and the poly (A) tail. It was about twice as effective as the full-length (FL) RNAe in some experiments (Figure 6B). Additionally we tested whether the SINEB2 could enhance mRNA translation on its own when placed on the 5'UTR of mRNA. We cloned either the SINEB2 sequence or its reverse complement sequence to the 5'UTR of an EGFP coding sequence, then transfected it into HEK293T cells. The results showed that neither reverse complement nor sense SINEB2 could enhance protein expression directly (Supplementary Figure S4A and B). It could thus be concluded that the minimal RNAe construct minRNAe only needs a pairing sequence to guide RNAe to the target mRNA which confers its specificity, a SINEB2 sequence which confers its enhancement function, and a poly (A) tail which provides the stability of this non-coding RNA.

#### The original lncRNA has alternative tailing

Further sequence analysis of the original RNAe sequence showed that a three-tailing-signal sequence AAUAAA was located downstream of the partial ALU sequence (Figure 6C). We chose two pairs of primers complementary to sequences upstream and downstream of the predicted alternative tailing signal, and measured the relative abundance of the amplified sequences by qRT-PCR. We found that the upstream part was about three times more abundant than the downstream (Figure 6D), which confirmed alternative tailing. Furthermore, sequencing of PCR products from 3' rapid amplification of cDNA ends (RACE) (Figure 6E) showed that only site 480 (Figure 6C) was utilized as the actual alternative tailing site.

### Possible applications of RNAe

#### RNAe showed its great advantages in a model of high-throughput screening

**Figure 7.**
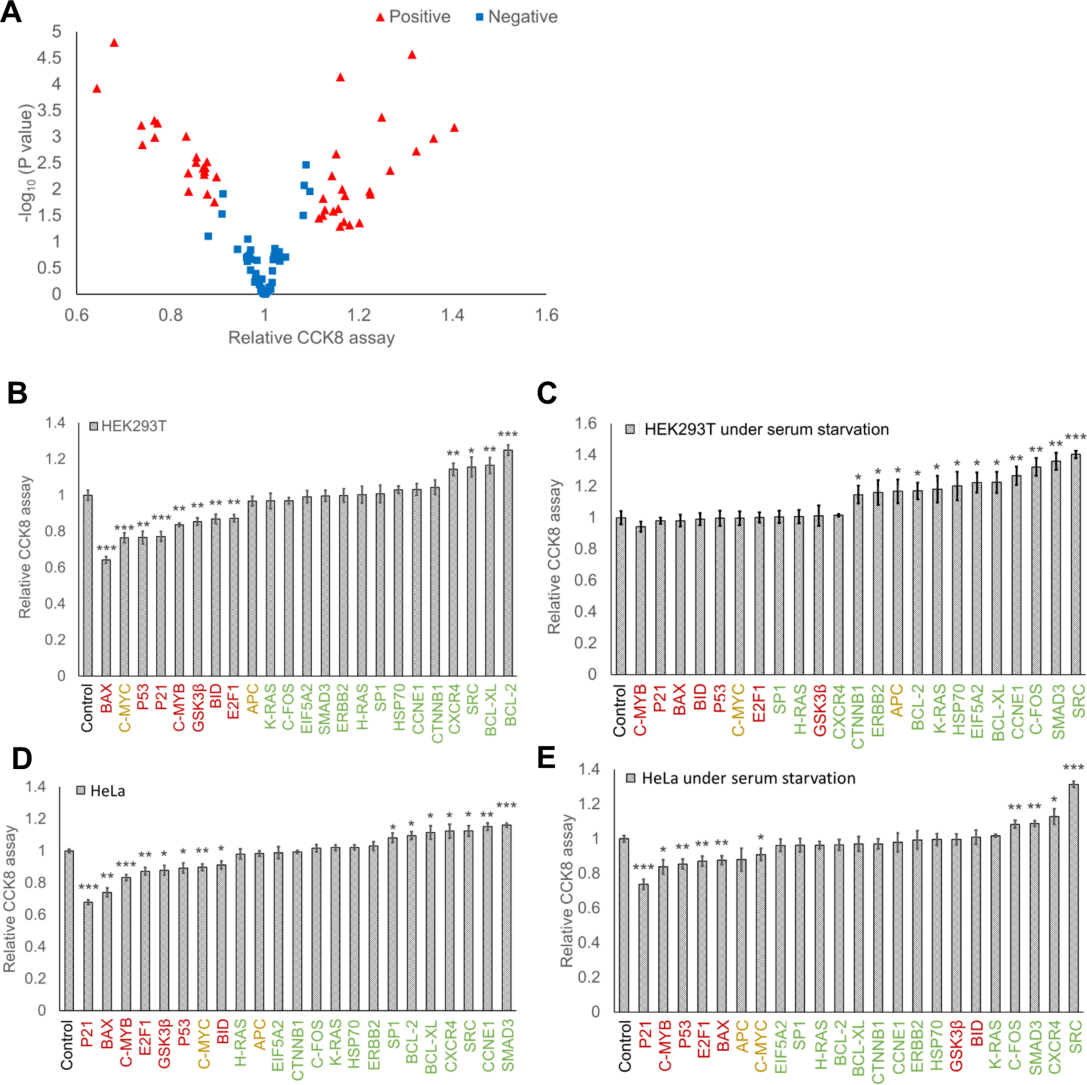
Application of RNAe in high-throughput screening. HEK293T or HeLa cells were transfected with RNAe constructs corresponding to the indicated genes and CCK8 assay was conducted to detect their influence on cell proliferation. (**A**) The effect of RNAe constructs on selected proliferation-associated genes in HEK293T and HeLa cells, as measured by their effect on proliferation detected by CCK8 assay. Red triangles represent positive genes with relative expression change > 10% and *P-*values < 0.05 in CCK8 blue squares represent genes deemed negative. (**B**–**E**) Respective effect of RNAe targeting of selected genes on proliferation in HEK293T cells with (C), or without serum starvation (B), or HeLa cells with (E) or without serum starvation (D). Green, red and yellow fonts indicate proliferative, anti-proliferative and controversial genes (genes that may affect cell proliferation in both directions depending on circumstances), respectively (mean ± SD, *n* = 3. *P < 0.05, **P < 0.01, ***P < 0.001).

Twenty three proliferation-associated genes (Supplementary Table S1) were chosen and tested with RNAe technology to validate the usage of RNAe as a screening tool (Figure 7). The corresponding RNAe plasmids (full-length series) were constructed and transfected into HEK293T or HeLa cells cultured with or without serum starvation. The effect of these RNAe constructs on cell proliferation was screened using the cell counting kit CCK8 (Figure 7A). Results showed that most of the proliferative genes (SMAD family member 3 (SMAD3), FBJ murine osteosarcoma viral oncogene homolog (C-FOS), v-src avian sarcoma (Schmidt-Ruppin A-2) viral oncogene homolog (SRC)), did promote cell growth, especially when the cells were starved of serum (Figure 7C and E). Conversely, most of the anti-proliferative genes (cyclin-dependent kinase inhibitor 1A (P21), BCL2-associated X protein (BAX), tumor protein p53 (P53), E2F transcription factor 1 (E2F1), BH3 interacting domain death agonist (BID), v-myb avian myeloblastosis viral oncogene homolog (C-MYB), glycogen synthase kinase 3 beta (GSK3β)) did inhibit cell growth, especially when cells were cultured in complete medium (Figure 7B and D).

**Figure 8.**
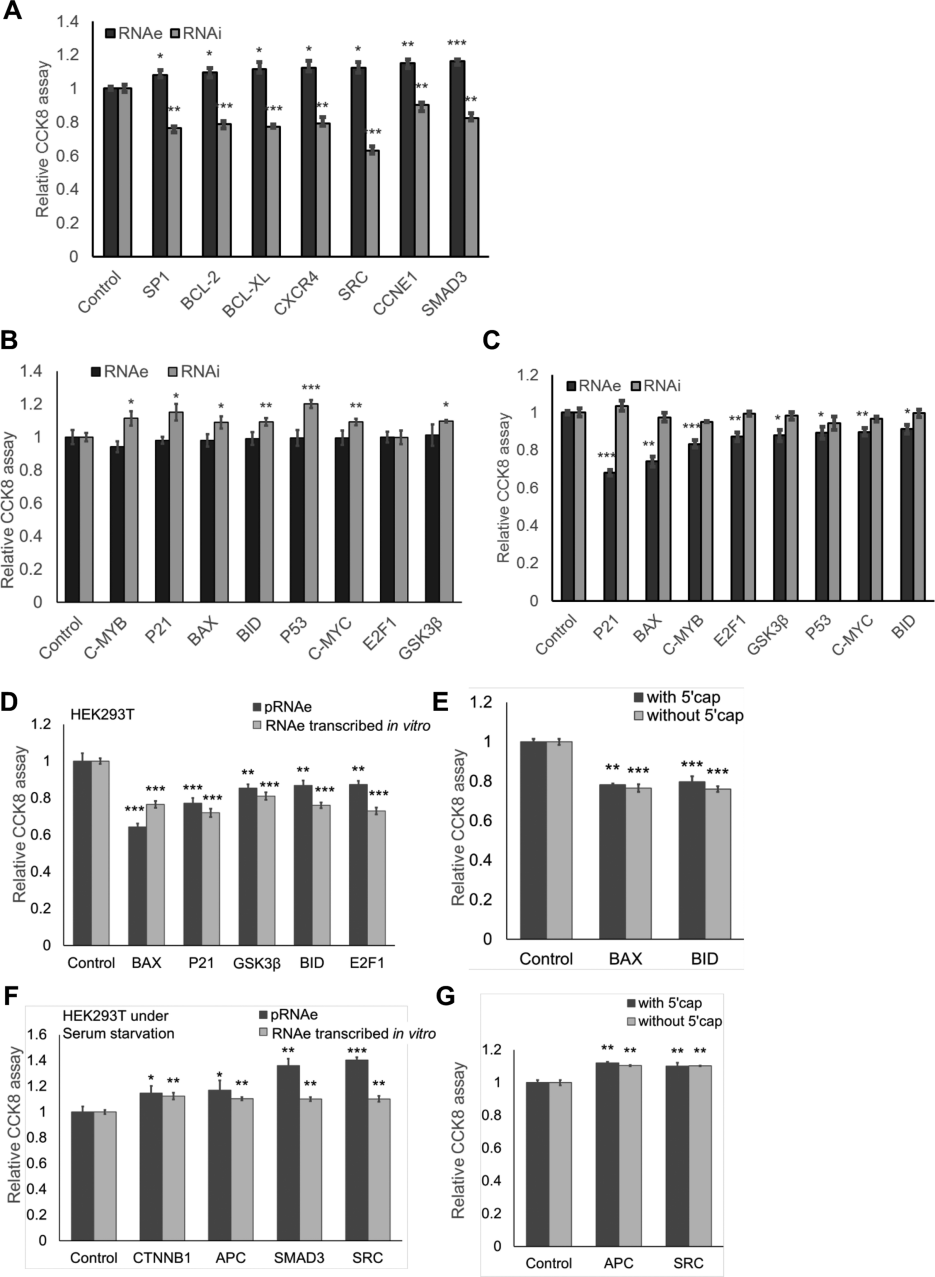
Extensive study of RNAe in high-throughput screening. HEK293T cells were transfected with RNAe and RNAi constructs corresponding to the indicated genes and CCK8 assay was conducted to detect their influence on cell proliferation. Results are shown according to the relative effects. (**A**) Comparison of RNAe and RNAi effect on genes with proliferative function. RNAe and RNAi both displayed significant effects. (**B** and **C**) Comparison of RNAe and RNAi effect on genes with anti-proliferative function under serum starvation (B) or normal conditions (C). In the group of anti-proliferative genes, RNAe had a significant effect under normal culture conditions (C), and RNAi under serum starvation (B). (**D** and **F**) RNAe constructs corresponding to the indicated genes were introduced into HEK293T calls either in the form of plasmid DNA (‘pRNAe’ group) or *in vitro* generated RNA (‘RNAe transcribed *in vitro*’ group, generated by *in vitro* transcription without 5' cap) under normal conditions (D) or under serum starvation (F), respectively. RNAe showed a significant effect when delivered in either form. (**E** and **G**) RNAe constructs transcribed *in vitro* with or without 5' cap (by adding m7G(5′)ppp(5′)G during *in vitro* transcription procedure) were introduced into cells under normal conditions (D) or serum starvation (F), respectively, and both methods of delivery had a similar effect (mean ± SD, *n* = 3. *P < 0.05, **P < 0.01, ***P < 0.001, two tailed *t*-test).

RNAe could also be demonstrated as equally effective and complementary to the well-known RNAi method. We proved that RNAe had the potential as a common high-throughput screening method for gene function screening. We used RNAi to confirm the accuracy of RNAe in high-throughput screening. We transfected RNAe plasmids targeting each of the seven genes with proliferative effects, and the corresponding RNAi constructs individually into HEK293T cells. We also conducted a parallel set of experiments using RNAi constructs for the respective target genes transfected into HEK293T cells as control. The results of RNAi screening were complementary to the RNAe results and opposite regarding their proliferative/anti-proliferative effect, respectively (Figure 8A). We then chose a different set of eight genes with anti-proliferative effects and compared the RNAi and RNAe results in the same way. We were somewhat surprised to find that RNAe had a marked effect even on genes which are silenced under natural circumstance, such as genes which induce apoptosis (Figure 8B and C). We further showed that RNAe can also be delivered into the target cells in the form of RNA, such as can be generated by *in vitro* transcription. These *in vitro* transcription experiments indicate that RNAe also offers advantages equal to those of RNAi such as the possibility of expression directly in the cytosol. This makes for improved transfection efficiency since plasmid entry into the nucleus is not necessary. We chose nine genes that are known to affect cell proliferation, and when tested as described above, four genes promoted and the other five inhibited cell proliferation. RNAs of the corresponding RNAe constructs were transcribed with a 5'cap *in vitro* and transfected into HEK293T cells. Plasmids with the same RNAe expression cassettes were used as control. The results showed that transfection of RNAe transcribed *in vitro* yielded the same effect as transfection of plasmid DNA with subsequent transcription *in vivo* (Figure 8D and F). Further experiments proved that RNAe can work even without the 5' cap. For this we chose four genes affecting cell proliferation. RNAe constructs were transcribed with or without a 5'cap and transfected into HEK293T cells. The 5'cap appears to have no influence on RNAe enhancement (Figure 8E and G). Taken as a whole, these results reveal that RNAe can be an effective tool for the screening of functional genes by enhancing target protein expression and demonstrate its efficiency, suitability for various delivery methods and complementarity to the well-established RNAi method.

#### Construction of pRNAe-Plus, a series of universal RNAe vectors for enhanced expression of heterologous eukaryotic proteins

**Figure 9.**
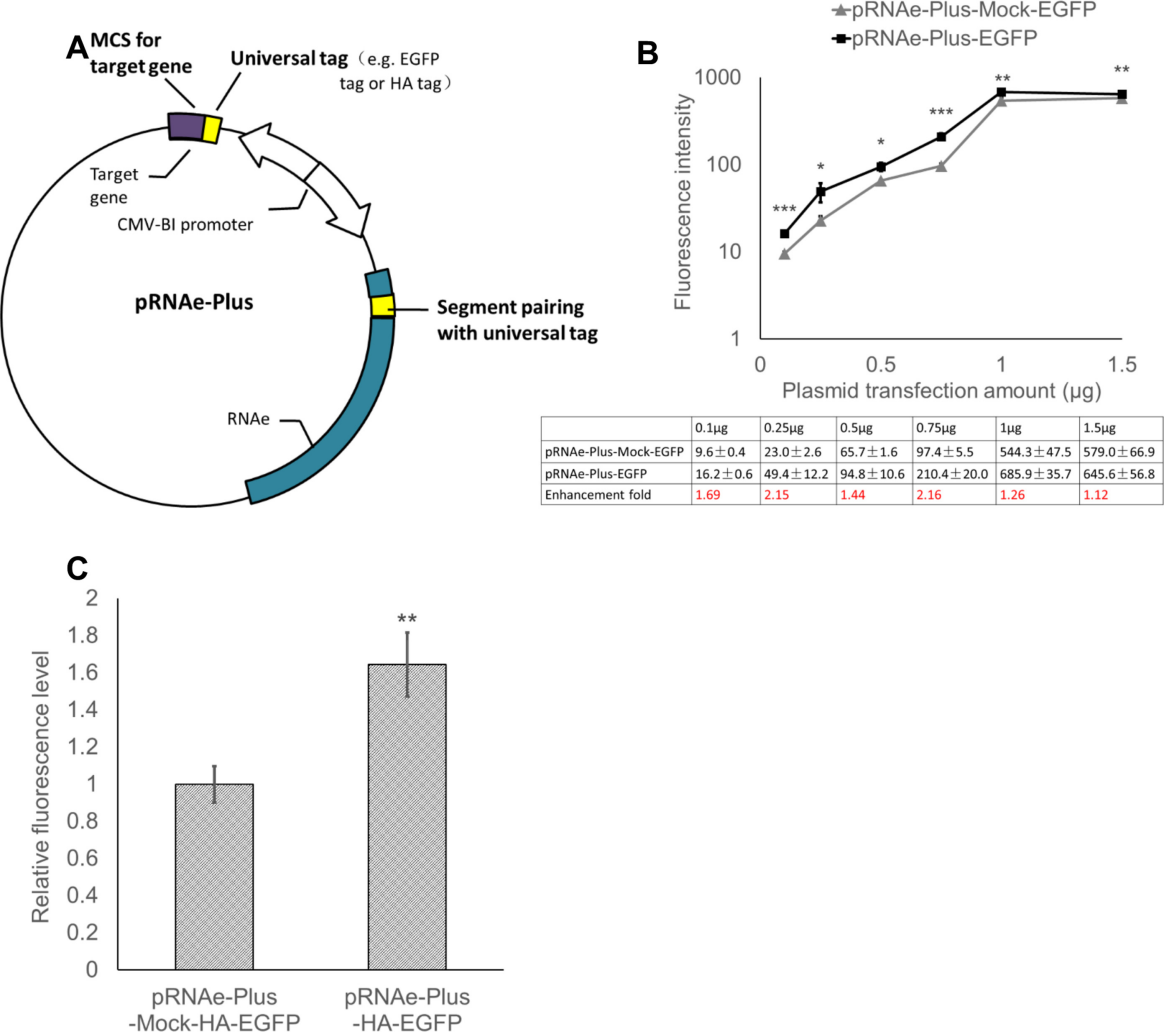
Construction of ‘pRNAe-Plus’ plasmids. (**A**–**C**) The construction (A) and effect on EGFP expression measured by fluorescence of the pRNAe-Plus with N-terminal EGFP-tag (B) and HA-tag (C). pRNAe-Plus-EGFP or pRNAe-Plus-Mock-EGFP were transfected to HEK293T cells using the amounts (per well of 12-well plates) indicated in the figure. pRNAe-Plus-HA-EGFP or pRNAe-Plus-Mock-HA-EGFP were transfected to HEK293T cells, respectively, at 1 μg per well of 12-well plates (mean ± SD, *n* = 3. *P < 0.05, **P < 0.01, ***P < 0.001, two tailed *t*-test).

We constructed a series of plasmid vectors termed pRNAe-Plus, each of which carries a ready-made matching RNAe in an expression plasmid capable of accepting a cDNA insert, designed to efficiently express a heterologous fusion protein. For example, the pRNAe-Plus-EGFP plasmid has a multiple-cloning site (MCS) downstream of the N-terminal EGFP tag and the RNAe pairs with the EGFP tag, with the expression driven individually by bi-directional CMV promoter (Figure 9A). This pRNAe-Plus-EGFP plasmid expressed EGFP significantly better than the control plasmid pRNAe-Plus-Mock-EGFP, which lacks the RNAe element but is otherwise identical. This was true over a dosage range from 0.1 μg to 1.5 μg of plasmid DNA (Figure 9B). A greater enhancement effect was seen at lower doses, which indicates a possible saturation of translation at higher DNA dosages. An expression plasmid encoding an N-terminal HA tag which is used widely in eukaryotic protein production has also been constructed. We did the same comparison of pRNAe-Plus HA-EGFP with its control plasmid pRNAe-Plus-Mock-HA-EGFP, and the results showed that the pRNAe-Plus design also worked well for the HA tag (Figure 9C). We expect that this construction scheme of pRNAe-Plus could be used to construct further expression plasmids with various N-terminal tags useful in eukaryotic protein production and purification.

#### RNAe can be used for eukaryotic antibody production

**Figure 10.**
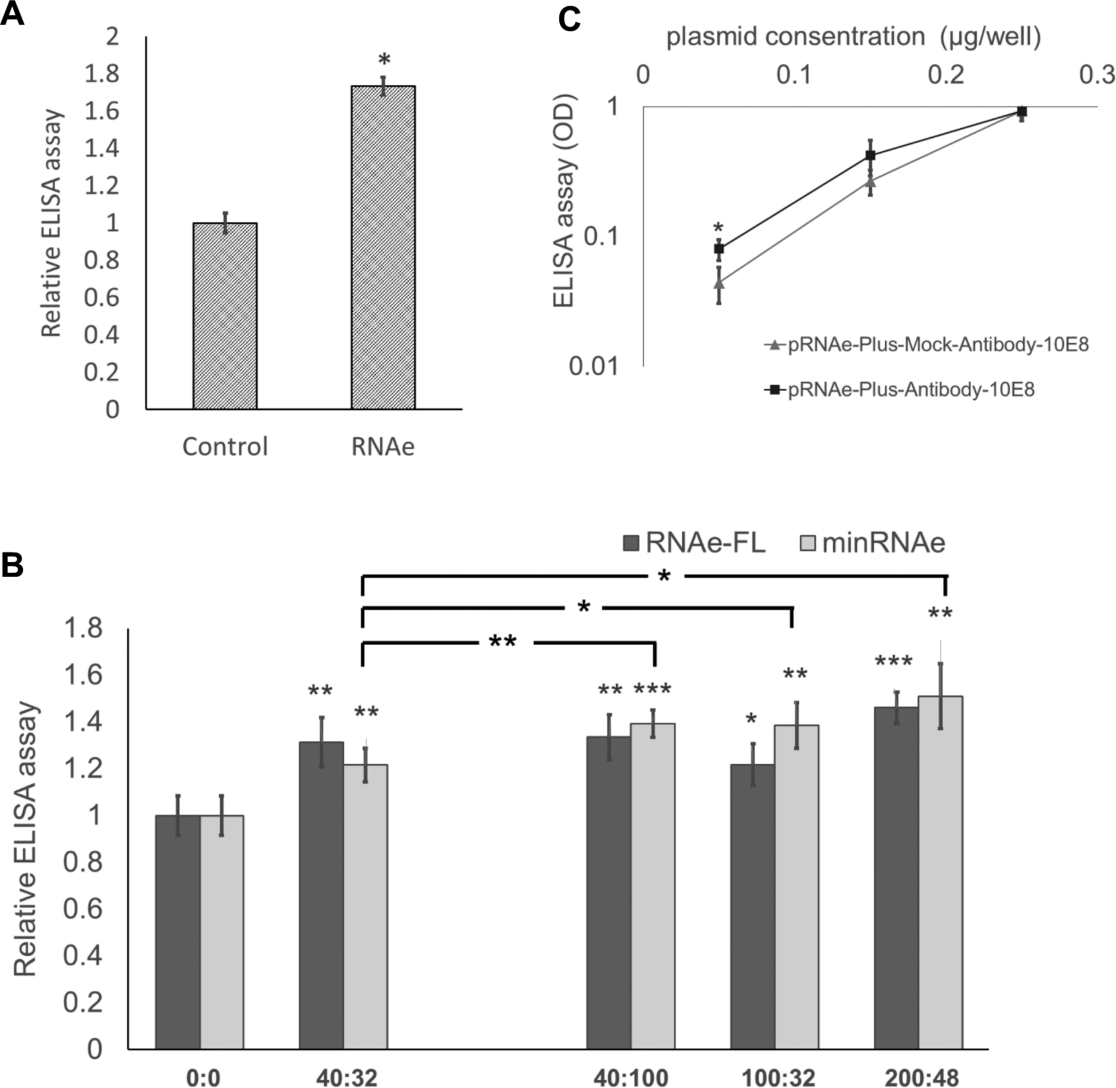
Application of RNAe in antibody production. (**A**) Effect of RNAe on the expression of anti-HIV antibody 10E8 in HEK293T cells co-transfected with 0.2 μg of pHIV-Antibody-10E8-H and pHIV-Antibody-10E8-L each and either 1.2 μg of pRNAe-Mock plasmid (Control group) or 1.2 μg of pRNAe-Ab-UNI-40:32-FL plasmid (RNAe group) (mean ± SD, *n* = 6. *P < 0.05, two tailed *t*-test). (**B**) Optimization of pairing sequence increased the enhancement effect of RNAe on anti-HIV antibody 10E8 production (mean ± SD, *n* = 6. *P < 0.05, two tailed *t*-test). (**C**) The effect of pRNAe-Plus containing an antibody signal sequence on antibody production. Either pRNAe-Plus-Mock-HIV-Antibody-10E8-H and pRNAe-Plus-Mock-HIV-Antibody-10E8-L (control group) or pRNAe-Plus-HIV-Antibody-10E8-H and pRNAe-Plus-HIV-Antibody-10E8-L (pRNAe-Plus group) were co-transfected into HEK293T cells, respectively, using DNA amounts as indicated in the figure and their effect on antibody production as measured by ELISA (mean ± SD, *n* = 6. *P < 0.05, **P < 0.01, ***P < 0.001, two tailed *t*-test).

For a proof of principle of eukaryotic antibody production we used 10E8 (12), a monoclonal antibody against Human Immunodeficiency Virus (HIV) (Figure 10A). Since previous results showed that optimization for a specified protein may reach a better enhancement effect than the original 40:32 RNAe, a series of RNAe plasmids with different length RNAe constructs and corresponding minRNAe plasmids were constructed and tested for anti-HIV antibody 10E8 production. ELISA results showed that compared with the original RNAe, several choices showed better expression. This indicates that RNAe can be further optimized on a pre-application basis and might be suitable for future use in industrial protein production (Figure 10B).

Additionally, a pRNAe-Plus engineered plasmid was constructed with a novel signal peptide sequence as is found in a novel antibody expression plasmid reported by Tiller *et al*.(13), together with the corresponding RNAe. Again, a bi-directional CMV promoter was used to express the antibody mRNA and corresponding RNAe, and the pRNAe-Plus-Antibody plasmid of the anti-HIV antibody 10E8 was used to test the new engineered plasmid in antibody production. Without exception, pRNAe-Plus-Antibody showed better effect than control vector in anti-HIV antibody 10E8 production (Figure 10C).

## DISCUSSION

In the present study, we developed RNAe, an efficient and universally applicable *in vitro* protein expression enhancement tool based on antisense RNA comprising an inverted SINEB2 repeat element. This technology originates from its natural prototype RNA transcript, the uchl1 gene lncRNA, which Carrieri*et al*. found to enhance the translation of its corresponding sense mRNA via base-pairing and by recruiting multiple ribosomes via the functional element SINEB2. Carrieri *et al*. made an AS-GFP lncRNA by altering only the 72 nt pairing part of the lncRNA. The present work started from this lncRNA construct which we renamed RNAe-egfpc1 and made a series of optimization efforts. We systematically tested the sequence elements in the lncRNA and deleted the non-essential sequences, producing a 282 nt minimal RNAe sequence that contains only a 72 nt antisense sequence, a 167 nt inverted SINEB2 sequence and a poly (A) tail. This minimal RNAe works just as well or even better than the full-length RNAe in enhancing target protein expression under comparable conditions (Figure 6A and B). In addition, RNAe did not impair either mRNA generation (Figure 2C) or stability (Figure 3A). On the other hand, without a pairing sequence, neither inverted nor sense SINEB2 could enhance protein expression (Supplementary Figure S4A and B), indicating that SINEB2 could not complete the RNAe function in isolation of other components.

In this study, the RNAe tool was delivered into cells in the form of plasmid DNA and then produced inside cells. Such plasmid transfection could be widely used for *in vitro* applications. Additionally, the RNAe sequence element could potentially be incorporated into any viral vector platform for use in appropriate cell lines *in vitro* or even viral vector targeted cells and tissues *in vivo*.

An RNAe plasmid (or potentially an RNAe viral vector) could be introduced into cells to enhance the expression of an endogenous protein (such as SOX9 in Figure 4C and D and the 23 proliferation-associated proteins in Figure 7) or used in combination with another expression plasmid carrying the target protein, such as the EGFP, EGFP fusion proteins, and antibody heavy and light chains as used in the present study (Figures 2C, 4B and 10A). RNAe could also be incorporated into the very plasmid carrying its target gene, such as in the pRNAe-Plus platform shown in Figure 9.

The specificity of an RNAe could be very high as it matches its target mRNA both at its 5' untranslated and translated region (typically 40 nt 5' UTR and 32 nt 5' TR). For cytosolic proteins, the sequence is essentially unique as we have tried to search for sequences matching the pairing sequence of the proliferation-associated genes tested in Figure 7 in GenBank using the BLAST tool (14) and we found only a single high-match result with all other results having values below 50 (Supplementary Table S2). However, in cross-over experiments, RNAe still shows some off-target effects especially for genes with the same 5' UTR or 5' TR (Supplementary Figure S3), so it may not be suitable for individual targeting of different splicing isoforms or gene family members. For secreted proteins and engineered proteins, the specificity could vary depending on shared signal peptide and the designed parts of the plasmid sequence. On the other hand, this very feature might be useful. For example, since the antibody heavy and light chain mRNAs share the same signal peptide and 5' UTR, one RNAe construct could be used to enhance expression of a single antibody or a group of antibodies (pRNAe-Ab-UNI-40:32-FL can enhance both the heavy and light chain of antibody 10E8 as shown in Figure 10A), which is convenient and advantageous for *in vitro* production of antibodies.

The enhancement efficiency of RNAe appears to vary in different situations. In addition to dose effect (Figure 2C, Supplementary Figure S1), RNAe enhancement rate also varies for different cell lines and target proteins (Figure 4). The expression enhancements we have seen over the course of this study range from about 10-fold (1000%) down to a mere 10–20%. Regardless of these extremes, in general it appears quite routine to achieve a 50% or more enhancement with a typical RNAe (72 nt pairing sequence plus at least the SINEB2 element).

Thus, the RNAe tool has been added to the biotechnological protein production toolbox. Over the years many approaches and genetic elements have been explored and are now routinely used for the production of mammalian cell proteins, such as optimization of growth conditions for high efficient antibody production (15,16), the use of stronger promoters and enhancers to improve mRNA transcription level and thus gene expression (17), the addition of Kozak sequences to facilitate translational initiation (18) and codon optimization (19) for faster translation in appropriate cell systems. The RNAe technology acts at the post-transcriptional stage, apparently helping to recruit additional ribosomes to the target mRNA and increase translation density per mRNA. It is a new mechanism and enhances an aspect in protein expression unaddressed previously. It appears to work well with practically all existing protein engineering methods and provides significant expression enhancement at a reasonable dose. It can be anticipated that RNAe will be applied in the laboratory as well as in the industrial production of recombinant eukaryotic proteins, including antibodies.

Additionally, RNAe was successfully applied to high-throughput functional screening of proteins (Figure 7). A major advantage of RNAe is that it might make possible protein overexpression without gene cloning. Considering eukaryotic gene length and difficulty in cloning some very large or weakly expressed genes, RNAe technology may be a cost-effective and fast tool for high-throughput screening of functional genes. In addition, the functions of some knockdown-lethal genes, where RNAi cannot be used, can be better screened for using RNAe rather than cDNA cloning and overexpression. RNAe has the opposite effect to the widely-used RNAi on target protein but is otherwise comparable to RNAi in terms of delivery platform, labor intensity, speed, cost (20) and off-target rate (21). RNAe could also be combined with RNAi to regulate the expression of a target protein in both directions for functional study.

RNAe also has the potential to be used for gene therapy. Many genetic diseases such as some types of growth hormone deficiency (GHD) (22) or cystic fibrosis (CF) (23) are caused by single-copy-loss or promoter/enhancer mutations which lead to a decrease of protein level while the coding gene is still intact and present in the genome. For such diseases, RNAe holds a promise due to a likely tissue-specificity since it could enhance the target protein expression in a manner dependent on native mRNA distribution. RNAe also showed great simplicity compared with other tissue-specific methods (24–27).

In summary, RNAe technology may be a powerful new tool for protein expression enhancement in biological research and biopharmaceutical production.

## SUPPLEMENTARY DATA

Supplementary Data are available at NAR Online.

SUPPLEMENTARY DATA

## References

[1] Carrieri C., Cimatti L., Biagioli M., Beugnet A., Zucchelli S., Fedele S., Pesce E., Ferrer I., Collavin L., Santoro C. (2012). Long non-coding antisense RNA controls Uchl1 translation through an embedded SINEB2 repeat. Nature.

[2] Fan J.J., Martinez-Arguelles D.B., Papadopoulos V. (2011). Genome-wide expression of SINE B2-mediated natural antisense transcripts. FASEB J..

[3] Jabnoune M., Secco D., Lecampion C., Robaglia C., Shu Q., Poirier Y. (2013). A rice cis-natural antisense RNA acts as a translational enhancer for its cognate mRNA and contributes to phosphate homeostasis and plant fitness. Plant Cell.

[4] Pontier D.B., Gribnau J. (2011). Xist regulation and function explored. Hum. Genet..

[5] Modarresi F., Faghihi M.A., Lopez-Toledano M.A., Fatemi R.P., Magistri M., Brothers S.P., van der Brug M.P., Wahlestedt C. (2012). Inhibition of natural antisense transcripts *in vivo* results in gene-specific transcriptional upregulation. Nat. Biotechnol..

[6] Faghihi M.A., Zhang M., Huang J., Modarresi F., Van der Brug M.P., Nalls M.A., Cookson M.R., St-Laurent G., Wahlestedt C. (2010). Evidence for natural antisense transcript-mediated inhibition of microRNA function. Genome Biol.

[7] Wang Y., Pang W.J., Wei N., Xiong Y., Wu W.J., Zhao C.Z., Shen Q.W., Yang G.S. (2014). Identification, stability and expression of Sirt1 antisense long non-coding RNA. Gene.

[8] Ørom U.A., Nielsen F.C., Lund A.H. (2008). MicroRNA-10a binds the 5’UTR of ribosomal protein mRNAs and enhances their translation. Mol. Cell.

[9] Guo Q., Goto S., Chen Y., Feng B., Xu Y., Muto A., Himeno H., Deng H., Lei J., Gao N. (2013). Dissecting the *in vivo* assembly of the 30S ribosomal subunit reveals the role of RimM and general features of the assembly process. Nucleic Acids Res..

[10] Kawakami Y., Rodriguez-Leon J., Izpisua Belmonte J.C (2006). The role of TGFbetas and Sox9 during limb chondrogenesis. Curr. Opin. Cell Biol.

[11] Yao Y., He Y., Guan Q., Wu Q. (2014). A tetracycline expression system in combination with Sox9 for cartilage tissue engineering. Biomaterials.

[12] Yu Y., Tong P., Li Y., Lu Z., Chen Y. (2014). 10E8-like neutralizing antibodies against HIV-1 induced using a precisely designed conformational peptide as a vaccine prime. Sci. China C. Life Sci..

[13] Tiller T., Busse C.E., Wardemann H. (2009). Cloning and expression of murine Ig genes from single B cells. J. Immunol. Methods.

[14] Altschul S.F., Gish W., Miller W., Myers E.W., Lipman D.J. (1990). Basic local alignment search tool. J. Mol. Biol..

[15] Itoh Y., Ueda H., Suzuki E. (1995). Overexpression of bcl-2, apoptosis suppressing gene: prolonged viable culture period of hybridoma and enhanced antibody production. Biotechnol. Bioeng..

[16] Suzuki E., Ollis D.F. (1990). Enhanced antibody production at slowed growth rates: experimental demonstration and a simple structured model. Biotechnol. Progr..

[17] Wurm F., Bernard A. (1999). Large-scale transient expression in mammalian cells for recombinant protein production. Curr. Opin. Biotechnol..

[18] Kozak M. (1986). Point mutations define a sequence flanking the AUG initiator codon that modulates translation by eukaryotic ribosomes. Cell.

[19] Zolotukhin S., Potter M., Hauswirth W.W., Guy J., Muzyczka N. (1996). A “humanized” green fluorescent protein cDNA adapted for high-level expression in mammalian cells. J. Virol..

[20] Dorsett Y., Tuschl T. (2004). siRNAs: applications in functional genomics and potential as therapeutics. Nat. Rev. Drug Discov..

[21] Lin X., Ruan X., Anderson M.G., McDowell J.A., Kroeger P.E., Fesik S.W., Shen Y. (2005). siRNA-mediated off-target gene silencing triggered by a 7 nt complementation. Nucleic Acids Res..

[22] Kyriaki S.A., Mehul T.D. (2012). Phenotype-genotype correlations in congenital isolated growth hormone deficiency (IGHD). Indian J. Pediatr..

[23] Steven M.R., Stacey M., Eric J.S. (2005). Cystic Fibrosis. N. Engl. J. Med..

[24] Toscano M.G., Romero Z., Muñoz P., Cobo M., Benabdellah K., Martin F. (2011). Physiological and tissue-specific vectors for treatment of inherited diseases. Gene Ther..

[25] Chuah M.K., Petrus I., De Bleser P., Le Guiner C., Gernoux G., Adjali O., Nair N., Willems J., Evens H., Rincon M.Y. (2014). Liver-specific transcriptional modules identified by genome-wide in silico analysis enable efficient gene therapy in mice and non-human. Primates. Mol. Ther..

[26] Hinderer C., Bell P., Gurda B.L., Wang Q., Louboutin J. P., Zhu Y., Bagel J., O'Donnell P., Sikora T., Ruane T. (2014). Liver-directed gene therapy corrects cardiovascular lesions in feline mucopolysaccharidosis type I.

[27] Ravikanth D., Gopinath K., Kalaivani G., Uma M.K., Khetan V., Sailaja E., Nivedita C., Subramanian K. (2013). Targeted expression of suicide gene by tissue-specific promoter and microRNA regulation for cancer gene therapy. PLoS One.

