# Supplementary material for: RNAe: an effective method for targeted protein translation enhancement by artificial non-coding RNA with SINEB2 repeat
Source: Nucleic Acids Res. 2015 Feb 26;43(9):e58. doi: 10.1093/nar/gkv125 (PMC4482056; doi:10.1093/nar/gkv125)
Supplement: SUPPLEMENTARY DATA [file supp_43_9_e58__index.html]

RNAe: an effective method for targeted protein translation enhancement by artificial non-coding RNA with SINEB2 repeat — SUPPLEMENTARY DATA 

# RNAe: an effective method for targeted protein translation enhancement by artificial non-coding RNA with SINEB2 repeat

## SUPPLEMENTARY DATA

**Files in this Data Supplement:**

- Supplementary Figure 1
- Supplementary Table 1
- Supplementary Reference
