# Supplementary material for: RNAe: an effective method for targeted protein translation enhancement by artificial non-coding RNA with SINEB2 repeat
Source: Nucleic Acids Res. 2015 Feb 26;43(9):e58. doi: 10.1093/nar/gkv125 (PMC4482056; doi:10.1093/nar/gkv125)
Supplement: SUPPLEMENTARY DATA [file supp_gkv125_nar-02547-met-g-2014-File012.docx]

**
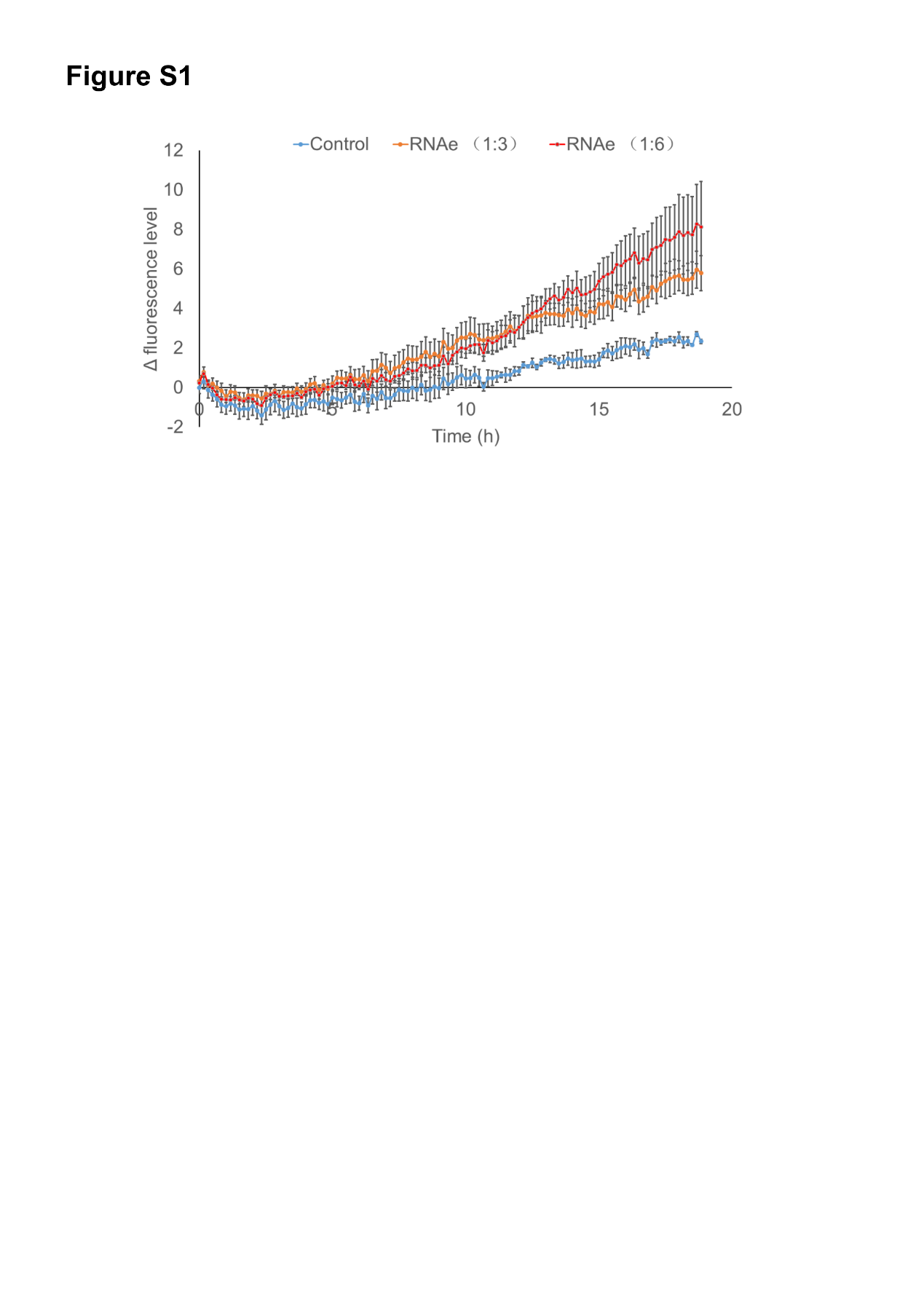
**

**Supplementary Figure 1** Fluorescence accumulation of continuously culturing HEK293T cells transfected with different dose of RNAe, by time-lapse assay. (mean ± s.d., n=4)


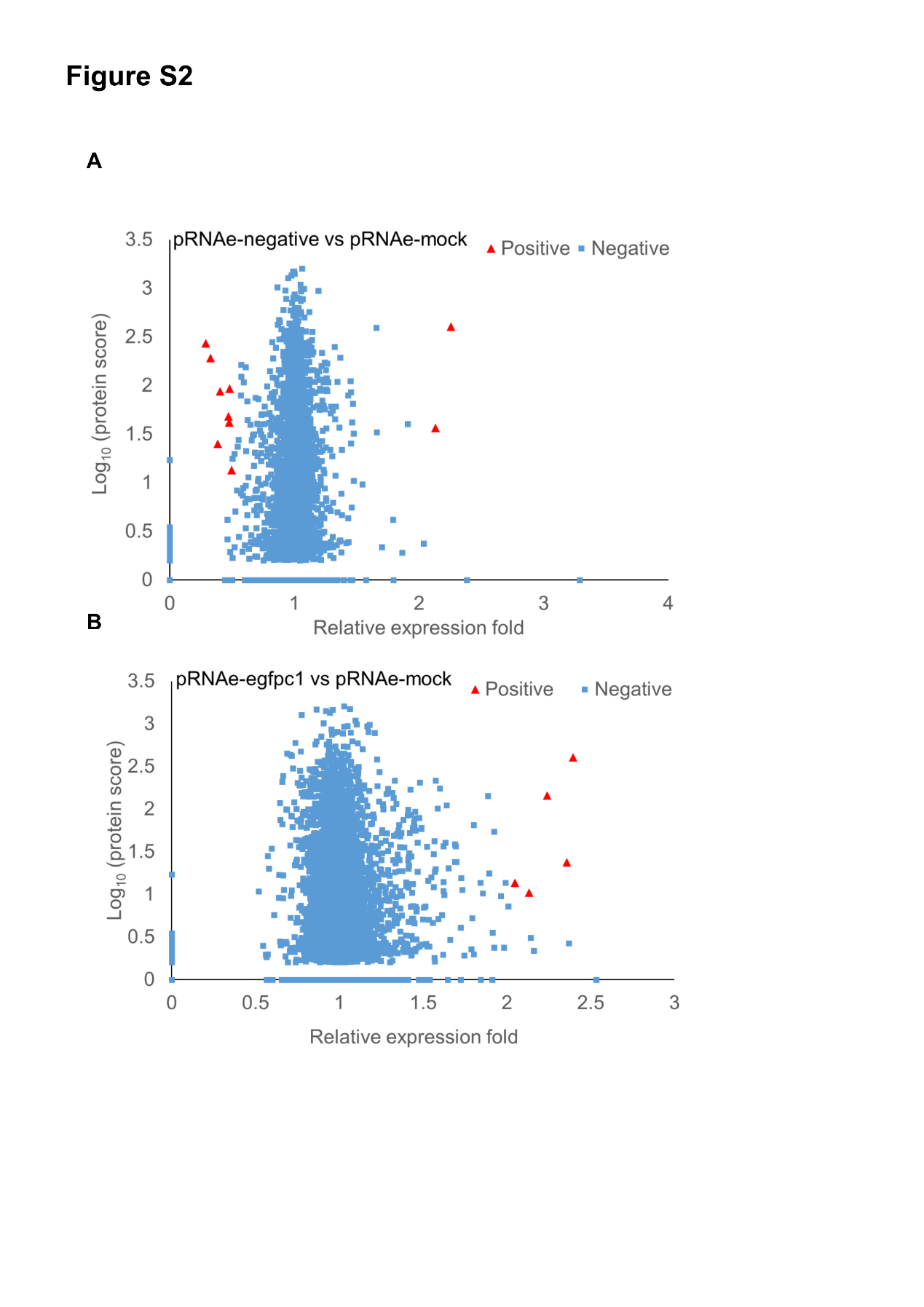


**Supplementary Figure 2** Specificity of RNAe ensured by proteomics analysis. A, B. RNAe-negative compared to RNAe-mock (A) and RNAe-egfpc1 compared to RNAe-mock (B) were shown. Red triangles stand for result-positive proteins with relative expression change > 50% and peptide score > 10 in quantitative mass spectrometry experiment, and the rest were result-negative genes marked by blue squares.


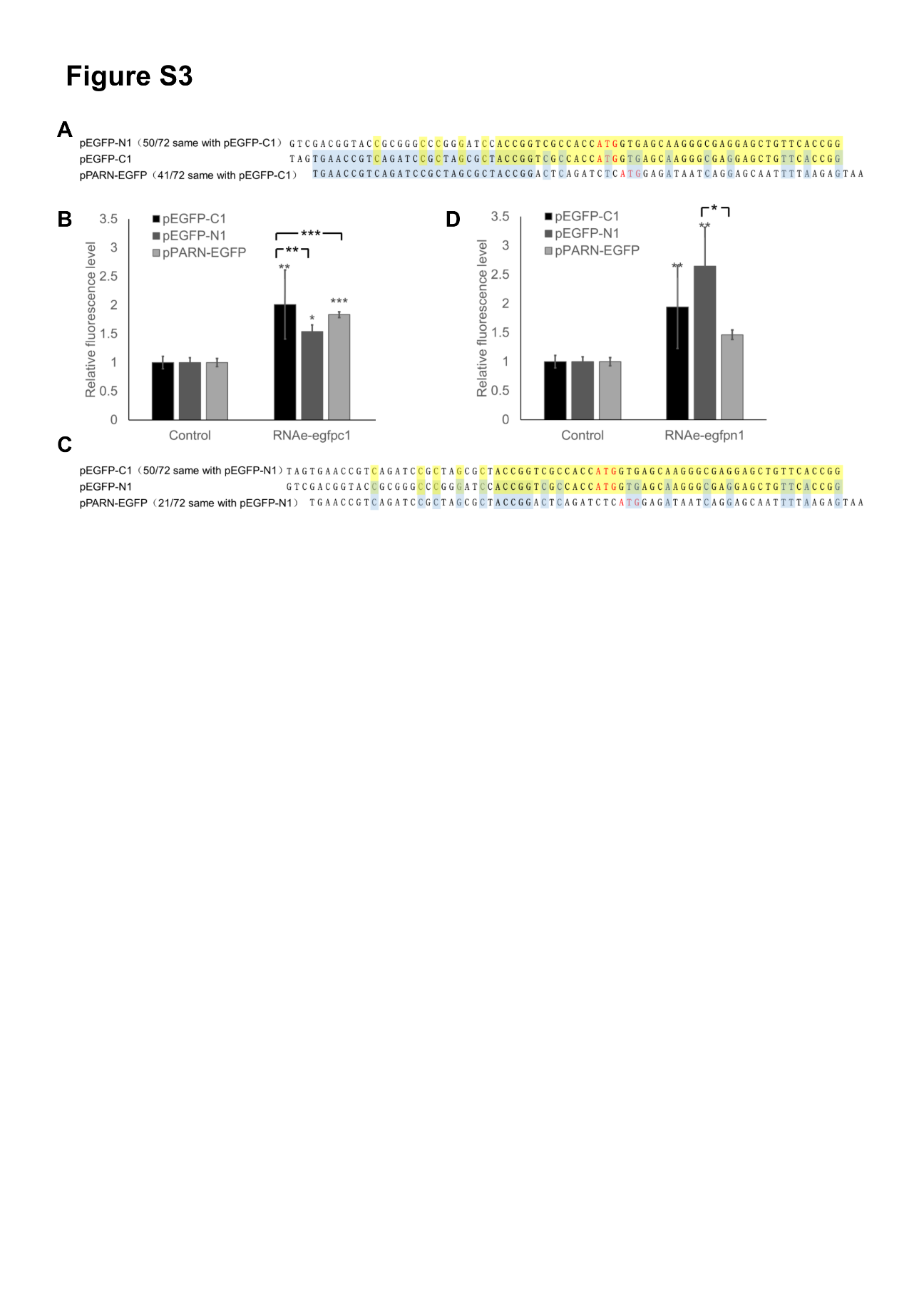


**Supplementary Figure 3** Side effect for RNAe to similar gene sequence with same 5’ TR or 5’ UTR. A. Sketch map of sequence difference between pEGFP-C1 and the rest two EGFP or EGFP fusion protein. 72 nt segments pairing with the corresponding RNAe are shown with the initiation codon indicated in red. pEGFP-C1 overlaps with 50 (yellow background) and 41 (blue background) nucleotides of pEGFP-N1 and pPARN-EGFP, respectively. B. Relative fluorescence strength reveals side effects of pRNAe-egfpc1 on expressions of the three proteins shown in A. C. Sketch map of sequence differences between pEGFP-N1 and the other EGFP or EGFP fusion proteins used int his study. 72 nt segments pairing with the corresponding RNAe are shown with the initiation codon indicated in red. pEGFP-N1 overlaps with 50 (yellow background) and 21 (blue background) nucleotides of pEGFP-C1 and pPARN-EGFP, respectively. D. Relative fluorescence strengths reveal side effect of pRNAe-egfpn1 to the three proteins shown in C. (mean ± s.d., n=4. * P < 0.05, ** P < 0.01, *** P < 0.001, two tailed t-test)


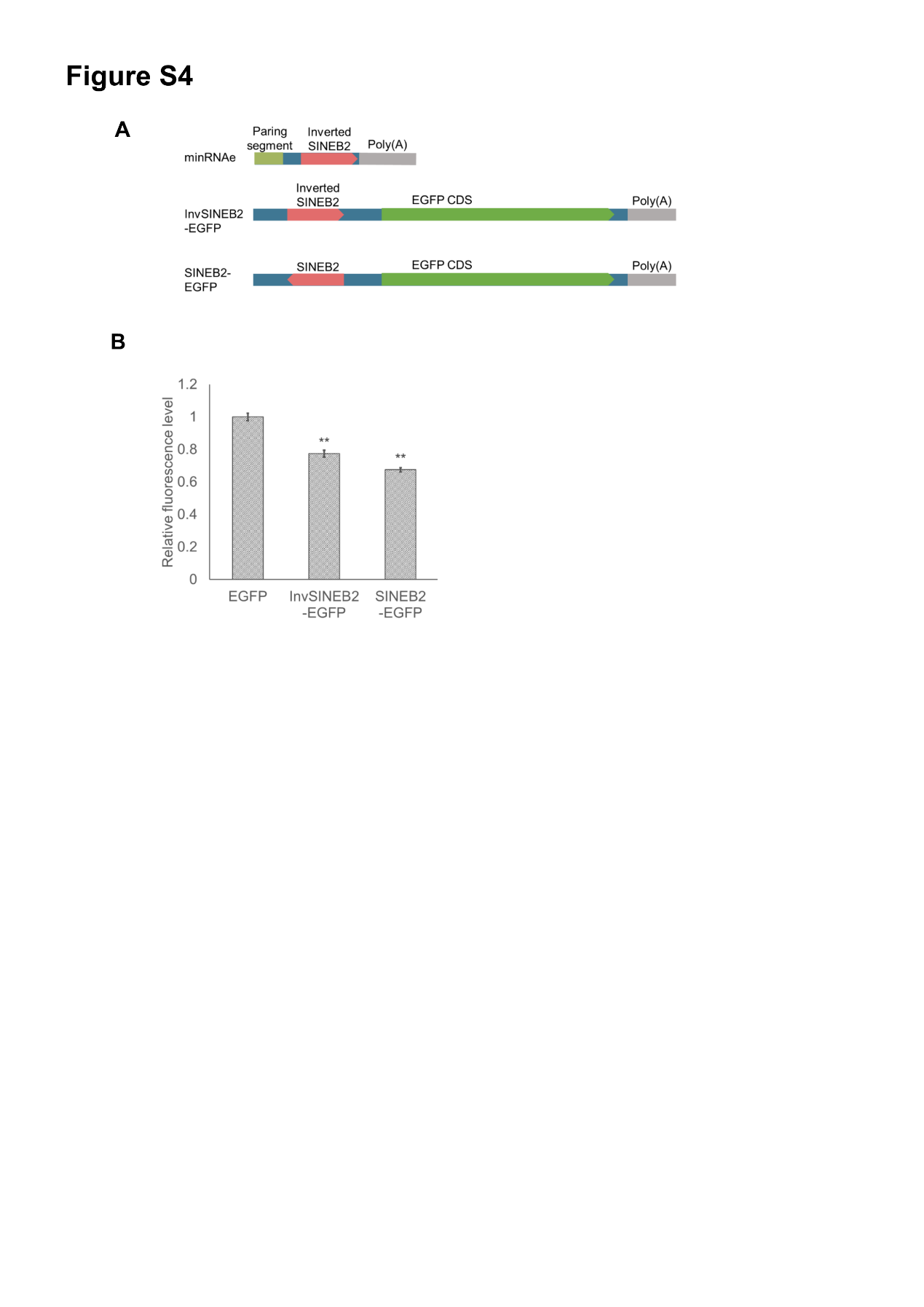


**Supplementary Figure 4** Sketch map (A) and effect (B) of SINEB2 sequence individually and directly on translation regulation. (mean ± s.d., n=4. ** P < 0.01, two tailed t-test)
