# Supplementary material for: RNAe: an effective method for targeted protein translation enhancement by artificial non-coding RNA with SINEB2 repeat
Source: Nucleic Acids Res. 2015 Feb 26;43(9):e58. doi: 10.1093/nar/gkv125 (PMC4482056; doi:10.1093/nar/gkv125)
Supplement: SUPPLEMENTARY DATA [file supp_gkv125_nar-02547-met-g-2014-File013.docx]

**Supplementary Table 1** Information of selected proliferation-associated genes for high-throughput screening

| Effect to proliferation | Gene Name | NCBI Accession | Reference |
| --- | --- | --- | --- |
| Proliferative | BCL-2 | NM_000633.2 | 1 |
|  | BCL-XL | Z23115.1 | 2 |
|  | CCNE1 | NM_001238.2 | 3 |
|  | C-FOS | V01512.1 | 4 |
|  | CTNNB1 | NM_001904.3 | 5 |
|  | CXCR4 | NM_001008540.1 | 6 |
|  | EIF5A2 | NM_020390.5 | 7 |
|  | ERBB2 | NM_004448.3 | 8 |
|  | H-RAS | AJ437024.1 | 9 |
|  | HSP70 | BC126122.1 | 10 |
|  | K-RAS | M54968.1 | 11 |
|  | SMAD3 | NM_005902.3 | 12 |
|  | SP1 | NM_138473.2 | 13 |
|  | SRC | NM_005417.4 | 14 |
| Inhibitive | BAX | NM_138764.4 | 1 |
|  | BID | NM_197966.2 | 15 |
|  | C-MYB | M15024.1 | 16 |
|  | E2F1 | NM_005225.2 | 17 |
|  | GSK3β | NM_002093.3 | 18 |
|  | P21 | NM_000389.4 | 19 |
|  | P53 | NM_000546.5 | 20 |
| Controversial | APC | NM_005883.2 | 5,21 |
|  | C-MYC | V00568.1 | 22, 23 |

**Supplementary Table 2** Prediction of potential off-target site of RNAe by Blast (NCBI, USA)

| Gene Name | Corresponding RNAe Pairing Segment Sequence | Score | | | | | |
| --- | --- | --- | --- | --- | --- | --- | --- |
|  |  | Original score | Mismatch score | | | | |
|  |  |  | 100-130 | 80-99 | 60-79 | 50-59 | 40-49 |
| APC | AGCTGCTCGTAGGGCGCCACGGAGCTCGCCATCTTCAGCTCCTGCAGCGTCCGCTTACGTGCAGCTGGGCTG | 131 | ---- | ---- | ---- | ---- | NLRX1 |
| BAX | CCGCCTCTGGGCTGCTCCCCGGACCCGTCCATCACCGCCGCTCCCGCCGCCGCCTCTCGCCGGGTCCGCGCG | 131 | ---- | ---- | ---- | ---- | KCNF1，NKX2-3 |
| BCL-2 | TTATCGTACCCTGTTCTCCCAGCGTGCGCCATCCTTCCCAGAGGAAAAGCAACGGGGGCCAACGGCACCTCT | 131 | ---- | ---- | ---- | ---- | ---- |
| BCL-XL | TCAACCACCAGCTCCCGGTTGCTCTGAGACATTTTTATAATAGGGATGGGCTCAACCAGTCCATTGTCCAAA | 131 | ---- | ---- | ---- | ---- | ---- |
| BID | CGAGCCATCATGACCCCAGCACCGCTGCACATTCGTATTTGTTGAATGAATGAATGAACCCTTGCCAGCCCA | 131 | ---- | ---- | ---- | ---- | FSHR |
| CCNE1 | TTCGCATCCCGCTCCCTGCGCTCCCTCGGCATGATGGGGCTGCTCCGGCCTGAGGCCAGGGTCTTGTCCGCG | 131 | ---- | ---- | ---- | ---- | LIPE |
| C-FOS | TCGTAGTCTGCGTTGAAGCCCGAGAACATCATCGTGGCGGTTAGGCAAAGCCGGGCGAGGGGCCGAGGGGCG | 131 | ---- | ---- | ---- | ---- | ---- |
| C-MYB | CTATATATGCTGTGCCGGGGTCTTCGGGCCATGGCGCGGCGGGCGGCGGGGCTCCGCCGAGAGCCGCGGGGA | 131 | ---- | ---- | ---- | ---- | ---- |
| C-MYC | TTCCTGTTGGTGAAGCTAACGTTGAGGGGCATCGTCGCGGGAGGCTGCTGGTTTTCCACTACCCGAAAAAAA | 131 | ---- | ---- | ---- | ---- | ---- |
| CTNNB1 | TCCAACTCCATCAAATCAGCTTGAGTAGCCATTGTCCACGCTGGATTTTCAAAACAGTTGTATGGTATACTT | 131 | ---- | ---- | ---- | ---- | ---- |
| CXCR4 | TATATCTGCAAAAGAGGCAAAGGAATGGACATTCACTTCCAATTCAGCAAGCATTAACCCAGTTAAAAAAAA | 131 | ---- | ---- | ---- | ---- | ---- |
| E2F1 | GGGCCGCCCGCAGGGGCCCCGGCCAAGGCCATGACGCTCACGGCCCGCGCGGCCCGGGTGACAGGCGGCGGC | 131 | ---- | ---- | ---- | ---- | KISS1R |
| EGFP-C1 | CCGGTGAACAGCTCCTCGCCCTTGCTCACCATGGTGGCGACCGGTAGCGCTAGCGGATCTGACGGTTCACTA | 131 | ---- | ---- | ---- | ---- | ---- |
| EIF5A2 | TCTCCAGTAGTGAAATCAATTTCGTCTGCCATGGTGGGCAGGGGAGATGGTAGTTTTTCCGTGGGAACTTTC | 131 | ---- | ---- | ---- | ---- | ---- |
| ERBB2 | AGCCCCCAGCGGCACAAGGCCGCCAGCTCCATGGTGCTCACTGCGGCTCCGGCCCCATGGCTCCGGCTGGAC | 131 | ---- | ---- | ---- | ---- | CAPN5 |
| GSK3β | GCAAAGGAGGTGGTTCTGGGCCGCCCTGACATGATCACTCTCTTCGCGAATCACCTTTTCCTTCCTTCCTCC | 131 | ---- | ---- | ---- | ---- | ---- |
| H-RAS | GCGCCCACCACCACCAGCTTATATTCCGTCATCGCTCCTCAGGGGCCTGCGGCCCGGGGTCCTCCTACAGGG | 131 | ---- | ---- | ---- | ---- | GATA5 |
| HSP70 | TGGAAGCCCAGGTCTATGCCCACCACCGACATGGCGCCGGCTACTGCTCGGGCTCGGGCCCGGGTCCGGCCA | 131 | ---- | ---- | ---- | ---- | HSPH1，STK10，NR2F1,PTPRF |
| K-RAS | GCTCCAACTACCACAAGTTTATATTCAGTCATTTTCAGCAGGCCTCTCTCCCGCACCTGGGAGCCGCTGAGC | 131 | ---- | ---- | ---- | ---- | ---- |
| P21 | TTCTGACGGACATCCCCAGCCGGTTCTGACATGGCGCCTCCTCTGAGTGCCTCGGTGCCTCGGCGAATCCGC | 131 | ---- | ---- | ---- | ---- | ---- |
| P53 | TCGACGCTAGGATCTGACTGCGGCTCCTCCATGGCAGTGACCCGGAAGGCAGTCTGGCTGCCAATCCAGGGA | 131 | ---- | ---- | ---- | ---- | ---- |
| SMAD3 | ATCGGGGGAGTGAAAGGCAGGATGGACGACATGGCTGGGGAGGGCGCGCGGGCGGCGAGGAGCGCCCCCGGC | 131 | ---- | ---- | ---- | ---- | SYDE2 |
| SP1 | ATTTCATCCATGGAGTGATCTTGGTCGCTCATGGTGGCAGCTGAGGGACAAGCTCAAGGGGGTCCTGTCCGG | 131 | ---- | ---- | ---- | ---- | ---- |
| SRC | GCATCCTTGGGCTTGCTCTTGTTGCTACCCATGGTCCTGGTAGAAGGCAGGGGCTGTCCCAGAGGACCTGGG | 131 | ---- | ---- | ---- | ---- | ---- |

**Supplementary Table 3** Plasmids used in the article

| Name | Source | Description | Cloning strategy |
| --- | --- | --- | --- |
| pSOX9 | named pCS9 in Yao et al.(24) | Vector for constitutive SOX9 expression | —— |
| pRNAe-egfpc1 | Named AS-GFP in Carrieri et al. (25) | Vector for RNAe targeting pEGFP-C1 expression | —— |
| pEGFP-N1 | Clonetech，USA | Vector for constitutive eGFP expression | —— |
| pEGFP-C1 | Clonetech，USA | Vector for constitutive eGFP expression | —— |
| pRNAe-mock | Named pN1 in Yao et al.[24] | Vector for negative control | —— |
| pRNAe-negative | This work | Vector for negative control | Relative pairing sequence of pRNAe-egfpc1 was deleted by digestion of Bsp120I/NotI and re-ligation |
| pRNAe-sox9 | This work | Vector for RNAe targeting endogenuous sox9 mRNA expression | Relative pairing sequence (GTCATCTTCATGAAGGGGTCCAGGAGATTCATACGCGAGCCCGGGGCAGGGGGCGGGTGGCCGGGAAAGGAG) was synthesized and cloned into pRNAe-egfpc1 with XhoI/NotI |
| pRNAe-Ab-UNI-40:32-FL | This work | Vector for RNAe targeting pHIV-Antibody-10E8-H&pHIV-Antibody-10E8-L with 40:32 expression | Relative pairing sequence (ACTAGAAAAAGGATGATACATGACCATCCCATGGTGGAATTCAATCGATAGAACCGAGGTGCAGTTGGACCT) was synthesized and cloned into pRNAe-egfpc1 with XhoI/NotI |
| pRNAe-egfpc1-Δ200 | This work | Vector for RNAe targeting pEGFP-C1 with truncation of Δ200 expression | Relative pairing sequence of pRNAe-egfpc1 was deleted by PCR using primers (CGCTGATCAGCCTCGACTG and AACATAGTCTTCTTGATCAAAGAAAGAAC) and re-ligation |
| pRNAe-egfpc1-Δ400 | This work | Vector for RNAe targeting pEGFP-C1 with truncation of Δ400 expression | Relative pairing sequence of pRNAe-egfpc1 was deleted by PCR using primers (CGCTGATCAGCCTCGACTG and CTCCTGCACCTTGTTCACTTTTC) and re-ligation |
| pRNAe-egfpc1-Δ600 | This work | Vector for RNAe targeting pEGFP-C1 with truncation of Δ600 expression | Relative pairing sequence of pRNAe-egfpc1 was deleted by PCR using primers (CGCTGATCAGCCTCGACTG and AACTCATCGGTTCAATGGAAGTC) and re-ligation |
| pRNAe-egfpc1-Δ827 | This work | Vector for RNAe targeting pEGFP-C1 with truncation of Δ827 expression | Relative pairing sequence of pRNAe-egfpc1 was deleted by PCR using primers (CGCTGATCAGCCTCGACTG and TATCTCCCAGTCAGGCAATCCT) and re-ligation |
| pminRNAe-egfpc1 | This work | Vector for RNAe targeting pEGFP-C1 with truncation of SINEB2 expression | Relative pairing sequence of pRNAe-egfpc1 was deleted by PCR using primers (CGCTGATCAGCCTCGACTG and ACTGGAGCTAAAGAGATGGCTCA) and re-ligation |
| pRNAe-egfpn1-100:200 | This work | Vector for RNAe targeting pEGFP-N1 with 100:100 pairing expression | Relative pairing sequence (TAGGTCAGGGTGGTCACGAGGGTGGGCCAGGGCACGGGCAGCTTGCCGGTGGTGCAGATGAACTTCAGGGTCAGCTTGCCGTAGGTGGCATCGCCCTCGCCCTCGCCGGACACGCTGAACTTGTGGCCGTTTACGTCGCCGTCCAGCTCGACCAGGATGGGCACCACCCCGGTGAACAGCTCCTCGCCCTTGCTCACCATGGTGGCGACCGGTGGATCCCGGGCCCGCGGTACCGTCGACTGCAGAATTCGAAGCTTGAGCTCGAGATCTGAGTCCGGTAGCGCTAGCGGATCTGACGGT) was synthesized and cloned into pRNAe-egfpc1 with XhoI/NotI |
| pRNAe-egfpn1-0:200 | This work | Vector for RNAe targeting pEGFP-N1 with 0:200 pairing expression | Relative pairing sequence (TAGGTCAGGGTGGTCACGAGGGTGGGCCAGGGCACGGGCAGCTTGCCGGTGGTGCAGATGAACTTCAGGGTCAGCTTGCCGTAGGTGGCATCGCCCTCGCCCTCGCCGGACACGCTGAACTTGTGGCCGTTTACGTCGCCGTCCAGCTCGACCAGGATGGGCACCACCCCGGTGAACAGCTCCTCGCCCTTGCTCACCAT) was synthesized and cloned into pRNAe-egfpc1 with XhoI/NotI |
| pRNAe-egfpn1-100:100 | This work | Vector for RNAe targeting pEGFP-N1 with 40:200 pairing expression | Relative pairing sequence (CCTCGCCGGACACGCTGAACTTGTGGCCGTTTACGTCGCCGTCCAGCTCGACCAGGATGGGCACCACCCCGGTGAACAGCTCCTCGCCCTTGCTCACCATGGTGGCGACCGGTGGATCCCGGGCCCGCGGTACCGTCGACTGCAGAATTCGAAGCTTGAGCTCGAGATCTGAGTCCGGTAGCGCTAGCGGATCTGACGGT) was synthesized and cloned into pRNAe-egfpc1 with XhoI/NotI |
| pRNAe-egfpn1-40:100 | This work | Vector for RNAe targeting pEGFP-N1 with 40:100 pairing expression | Relative pairing sequence (CCTCGCCGGACACGCTGAACTTGTGGCCGTTTACGTCGCCGTCCAGCTCGACCAGGATGGGCACCACCCCGGTGAACAGCTCCTCGCCCTTGCTCACCATGGTGGCGACCGGTGGATCCCGGGCCCGCGGTACCGTCGAC) was synthesized and cloned into pRNAe-egfpc1 with XhoI/NotI |
| pRNAe-egfpn1-0:100 | This work | Vector for RNAe targeting pEGFP-N1 with 0:100 pairing expression | Relative pairing sequence (CCTCGCCGGACACGCTGAACTTGTGGCCGTTTACGTCGCCGTCCAGCTCGACCAGGATGGGCACCACCCCGGTGAACAGCTCCTCGCCCTTGCTCACCAT) was synthesized and cloned into pRNAe-egfpc1 with XhoI/NotI |
| pRNAe-egfpn1-100:32 | This work | Vector for RNAe targeting pEGFP-N1 with 100:32 pairing expression | Relative pairing sequence (CCGGTGAACAGCTCCTCGCCCTTGCTCACCATGGTGGCGACCGGTGGATCCCGGGCCCGCGGTACCGTCGACTGCAGAATTCGAAGCTTGAGCTCGAGATCTGAGTCCGGTAGCGCTAGCGGATCTGACGGT) was synthesized and cloned into pRNAe-egfpc1 with XhoI/NotI |
| pRNAe-egfpn1 | This work | Vector for RNAe targeting pEGFP-N1 expression | Relative pairing sequence (CCGGTGAACAGCTCCTCGCCCTTGCTCACCATGGTGGCGACCGGTGGATCCCGGGCCCGCGGTACCGTCGAC) was synthesized and cloned into pRNAe-egfpc1 with XhoI/Not |
| pRNAe-egfpn1-0:32 | This work | Vector for RNAe targeting pEGFP-N1 with 0:32 pairing expression | Relative pairing sequence (CCGGTGAACAGCTCCTCGCCCTTGCTCACCAT) was synthesized and cloned into pRNAe-egfpc1 with XhoI/NotI |
| pRNA -gfpn1-100:0 | This work | Vector for RNAe targeting pEGFP-N1 with 100:0 pairing expression | Relative pairing sequence (GGTGGCGACCGGTGGATCCCGGGCCCGCGGTACCGTCGACTGCAGAATTCGAAGCTTGAGCTCGAGATCTGAGTCCGGTAGCGCTAGCGGATCTGACGGT) was synthesized and cloned into pRNAe-egfpc1 with XhoI/NotI |
| pRNA  -gfpn1-40:0 | This work | Vector for RNAe targeting pEGFP-N1 with 40:0 pairing expression | Relative pairing sequence (GGTGGCGACCGGTGGATCCCGGGCCCGCGGTACCGTCGAC) was synthesized and cloned into pRNAe-egfpc1 with XhoI/NotI |
| pRNAe-egfpc1-40:360 | This work | Vector for RNAe targeting pEGFP-C1 with 40:360 pairing expression | Relative pairing sequence was cloned from pEGFP-C1 by primers (attattgcggccgcatcTAGTGAACCGTCAGATCCGCTAG and attatctcgagCAGGGTGTCGCCCTCGAA) and cloned into pRNAe-egfpc1 with XhoI/NotI |
| pRNAe-egfpc1-40:560 | This work | Vector for RNAe targeting pEGFP-C1 with 40:560 pairing expression | Relative pairing sequence was cloned from pEGFP-C1 by primers (attattgcggccgcatcTAGTGAACCGTCAGATCCGCTAG and attatctcgagGTGTTCTGCTGGTAGTGGTCGG) and cloned into pRNAe-egfpc1 with XhoI/NotI |
| pRNAe-egfpc1-40:760 | This work | Vector for RNAe targeting pEGFP-C1 with 40:760 pairing expression | Relative pairing sequence was cloned from pEGFP-C1 by primers (attattgcggccgcatcTAGTGAACCGTCAGATCCGCTAG and attatctcgagCGACTGCAGAATTCGAAGCTTG) and cloned into pRNAe-egfpc1 with XhoI/NotI |
| pEGFP-βB2-crystalline | in Zhang et al. (26) | Vector for constitutive EGFP-βB2-crystalline expression | —— |
| pPARN-EGFP | This work | Vector for constitutive PARN-EGFP expression | cDNA of PARN was cloned into pEGFP-N1 with SalI/BglII |
| pEGFP-BBCK | This work | Vector for constitutive EGFP-BBCK expression | cDNA of PARN was cloned into pEGFP-C1 |
| pRNAe-parn-egfp | This work | Vector for RNAe targeting pPARN-EGFP expression | Relative pairing sequence (TTACTCTTAAAATTGCTCCTGATTATCTCCATGAGATCTGAGTCCGGTAGCGCTAGCGGATCTGACGGTTCA) was synthesized and cloned into pRNAe-egfpc1 with XhoI/NotI |
| pMET-LUCIFERASE | This work | Vector for constitutive MET-LUCIFERASE expression | MET-LUCIFERASE was synthesized and cloned into pEGFP-C1 with AgeI/BglII |
| pRNAe-metluciferase | This work | Vector for RNAe targeting pMET-LUCIFERASE expression | Relative pairing sequence (AACACCAGGGTGAACACCACCTTGATGTCCATGGTGGCGACCGGTAGCGCTAGCGGATCTGACGGTTCACTA) was synthesized and cloned into pRNAe-egfpc1 with XhoI/NotI |
| pHIV-Antibody-10E8-H | in Yu et al. (27) | Vector for constitutive heavy chain of HIV-Antibody-10E8 expression | —— |
| pHIV-Antibody-10E8-L | in Yu et al. (27) | Vector for constitutive light chain of HIV-Antibody-10E8 expression | —— |
| pRNAe-Plus-HA-EGFP | This work | Vector of RNAe-Plus with HA-EGFP | Whole plasmid synthesized |
| pInvSINEB2-EGFP | This work | Vector for constitutive InvSINEB2-EGFP expression | Inverted SINEB2 sequence with deletion of ATG sequence was synthesized and cloned into pEGFP-N1 |
| pSINEB2-EGFP | This work | Vector for constitutive SINEB2-EGFP expression | SINEB2 sequence with deletion of ATG sequence was synthesized and cloned into pEGFP-N1 |
| pRNAe-Plus-EGFP | This work | Vector of RNAe-Plus with N-terminal EGFP tag | Whole plasmid synthesized |
| pRNAe-Plus-Mock-EGFP | This work | Mock vector of RNAe-Plus with N-terminal EGFP tag | Whole plasmid synthesized |
| pRNAe-Plus-Mock-HA-EGFP | This work | Mock vector of RNAe-Plus with HA-EGFP | Whole plasmid synthesized |
| pRNAe-Ab-E8H-40:100-FL | This work | Vector for RNAe targeting pHIV-Antibody-10E8-H with 40:100 pairing expression | Relative pairing sequence (CAGGCTTCACCAAGCCTCCCCCAGACTCCACCAGCTGCACCTCAGAATGTACACCGGTTGCAGTTGCTACTAGAAAAAGGATGATACATGACCATCCCATGGTGGAATTCAATCGATAGAACCGAGGTGCAGTTGGACCT) was synthesized and cloned into pRNAe-egfpc1 with XhoI/NotI |
| pRNAe-Ab-E8L-40:100-FL | This work | Vector for RNAe targeting pHIV-Antibody-10E8-L with 40:100 pairing expression | Relative pairing sequence (CCAGGGCCACAGAGACACCAGTCTCCTGTGTCAGCTCATAGGAGGTCACAGAACCGGTTGCAGTTGCTACTAGAAAAAGGATGATACATGACCATCCCATGGTGGAATTCAATCGATAGAACCGAGGTGCAGTTGGACCT) was synthesized and cloned into pRNAe-egfpc1 with XhoI/NotI |
| pRNAe-Ab-E8H-40:100-min | This work | Vector for minRNAe targeting pHIV-Antibody-10E8-H with 40:100 pairing expression | Relative pairing sequence (CAGGCTTCACCAAGCCTCCCCCAGACTCCACCAGCTGCACCTCAGAATGTACACCGGTTGCAGTTGCTACTAGAAAAAGGATGATACATGACCATCCCATGGTGGAATTCAATCGATAGAACCGAGGTGCAGTTGGACCT) was synthesized and cloned into pminRNAe-egfpc1 with XhoI/NotI |
| pRNAe-Ab-E8L-40:100-min | This work | Vector for minRNAe targeting pHIV-Antibody-10E8-L with 40:100 pairing expression | Relative pairing sequence (CCAGGGCCACAGAGACACCAGTCTCCTGTGTCAGCTCATAGGAGGTCACAGAACCGGTTGCAGTTGCTACTAGAAAAAGGATGATACATGACCATCCCATGGTGGAATTCAATCGATAGAACCGAGGTGCAGTTGGACCT) was synthesized and cloned into pminRNAe-egfpc1 with XhoI/NotI |
| pRNAe-Ab-UNI-200:48-FL | This work | Vector for RNAe targeting pHIV-Antibody-10E8-H&pHIV-Antibody-10E8-L with 200:48 pairing expression | Relative pairing sequence (ACCGGTTGCAGTTGCTACTAGAAAAAGGATGATACATGACCATCCCATGGTGGAATTCAATCGATAGAACCGAGGTGCAGTTGGACCTGGGAGTGGACACCTGTGGAGAGAAAGGCAAAGTGGATGTTATTCTATAGTGTCACCTAAATCGTATGTGTATGATACATAAGGTTATGTATTAATTGTAGCCGCGTTCTAACGAAGCCAAGGGGGTGGGCCTATAGACTCTATAGGCGGTACTTACGTCA) was synthesized and cloned into pRNAe-egfpc1 with XhoI/NotI |
| pRNAe-Ab-UNI-200:48-min | This work | Vector for minRNAe targeting pHIV-Antibody-10E8-H&pHIV-Antibody-10E8-L with 200:48 pairing expression | Relative pairing sequence (ACCGGTTGCAGTTGCTACTAGAAAAAGGATGATACATGACCATCCCATGGTGGAATTCAATCGATAGAACCGAGGTGCAGTTGGACCTGGGAGTGGACACCTGTGGAGAGAAAGGCAAAGTGGATGTTATTCTATAGTGTCACCTAAATCGTATGTGTATGATACATAAGGTTATGTATTAATTGTAGCCGCGTTCTAACGAAGCCAAGGGGGTGGGCCTATAGACTCTATAGGCGGTACTTACGTCA) was synthesized and cloned into pminRNAe-egfpc1 with XhoI/NotI |
| pRNAe-Ab-UNI-100:32-FL | This work | Vector for RNAe targeting pHIV-Antibody-10E8-H&pHIV-Antibody-10E8-L with 100:32 pairing expression | Relative pairing sequence (ACTAGAAAAAGGATGATACATGACCATCCCATGGTGGAATTCAATCGATAGAACCGAGGTGCAGTTGGACCTGGGAGTGGACACCTGTGGAGAGAAAGGCAAAGTGGATGTTATTCTAT) was synthesized and cloned into pRNAe-egfpc1 with XhoI/NotI |
| pRNAe-Ab-UNI-100:32-min | This work | Vector for minRNAe targeting pHIV-Antibody-10E8-H&pHIV-Antibody-10E8-L with 100:32 pairing expression | Relative pairing sequence (ACTAGAAAAAGGATGATACATGACCATCCCATGGTGGAATTCAATCGATAGAACCGAGGTGCAGTTGGACCTGGGAGTGGACACCTGTGGAGAGAAAGGCAAAGTGGATGTTATTCTAT) was synthesized and cloned into pminRNAe-egfpc1 with XhoI/NotI |
| pRNAe-Ab-UNI-40:32-min | This work | Vector for minRNAe targeting pHIV-Antibody-10E8-H&pHIV-Antibody-10E8-L with 40:32 pairing expression | Relative pairing sequence (ACTAGAAAAAGGATGATACATGACCATCCCATGGTGGAATTCAATCGATAGAACCGAGGTGCAGTTGGACCT) was synthesized and cloned into pminRNAe-egfpc1 with XhoI/NotI |
| pRNAe-gsk3β | This work | Vector for RNAe targeting endogenous gsk3β mRNA expression | Relative pairing sequence (GCAAAGGAGGTGGTTCTGGGCCGCCCTGACATGATCACTCTCTTCGCGAATCACCTTTTCCTTCCTTCCTCC) was synthesized and cloned into pRNAe-egfpc1 with XhoI/NotI |
| pRNAe-p21 | This work | Vector for RNAe targeting endogenous p21 mRNA expression | Relative pairing sequence (TTCTGACGGACATCCCCAGCCGGTTCTGACATGGCGCCTCCTCTGAGTGCCTCGGTGCCTCGGCGAATCCGC) was synthesized and cloned into pRNAe-egfpc1 with XhoI/NotI |
| pRNAe-e2f1 | This work | Vector for RNAe targeting endogenous e2f1 mRNA expression | Relative pairing sequence (GGGCCGCCCGCAGGGGCCCCGGCCAAGGCCATGACGCTCACGGCCCGCGCGGCCCGGGTGACAGGCGGCGGC) was synthesized and cloned into pRNAe-egfpc1 with XhoI/NotI |
| pRNAe-cxcr4 | This work | Vector for RNAe targeting endogenous cxcr4 mRNA expression | Relative pairing sequence (TATATCTGCAAAAGAGGCAAAGGAATGGACATTCACTTCCAATTCAGCAAGCATTAACCCAGTTAAAAAAAA) was synthesized and cloned into pRNAe-egfpc1 with XhoI/NotI |
| pRNAe-eif5a2 | This work | Vector for RNAe targeting endogenous eif5a2 mRNA expression | Relative pairing sequence (TCTCCAGTAGTGAAATCAATTTCGTCTGCCATGGTGGGCAGGGGAGATGGTAGTTTTTCCGTGGGAACTTTC) was synthesized and cloned into pRNAe-egfpc1 with XhoI/NotI |
| pRNAe-hsp70 | This work | Vector for RNAe targeting endogenous hsp70 mRNA expression | Relative pairing sequence (TGGAAGCCCAGGTCTATGCCCACCACCGACATGGCGCCGGCTACTGCTCGGGCTCGGGCCCGGGTCCGGCCA) was synthesized and cloned into pRNAe-egfpc1 with XhoI/NotI |
| pRNAe-smad3 | This work | Vector for RNAe targeting endogenous smad3 mRNA expression | Relative pairing sequence (ATCGGGGGAGTGAAAGGCAGGATGGACGACATGGCTGGGGAGGGCGCGCGGGCGGCGAGGAGCGCCCCCGGC) was synthesized and cloned into pRNAe-egfpc1 with XhoI/NotI |
| pRNAe-c-fos | This work | Vector for RNAe targeting endogenous c-fos mRNA expression | Relative pairing sequence (TCGTAGTCTGCGTTGAAGCCCGAGAACATCATCGTGGCGGTTAGGCAAAGCCGGGCGAGGGGCCGAGGGGCG) was synthesized and cloned into pRNAe-egfpc1 with XhoI/NotI |
| pRNAe-cnne1 | This work | Vector for RNAe targeting endogenous ccne1 mRNA expression | Relative pairing sequence (TTCGCATCCCGCTCCCTGCGCTCCCTCGGCATGATGGGGCTGCTCCGGCCTGAGGCCAGGGTCTTGTCCGCG) was synthesized and cloned into pRNAe-egfpc1 with XhoI/NotI |
| pRNAe-bid | This work | Vector for RNAe targeting endogenous bid mRNA expression | Relative pairing sequence (CGAGCCATCATGACCCCAGCACCGCTGCACATTCGTATTTGTTGAATGAATGAATGAACCCTTGCCAGCCCA) was synthesized and cloned into pRNAe-egfpc1 with XhoI/NotI |
| pRNAe-k-ras | This work | Vector for RNAe targeting endogenous k-ras mRNA expression | Relative pairing sequence (GCTCCAACTACCACAAGTTTATATTCAGTCATTTTCAGCAGGCCTCTCTCCCGCACCTGGGAGCCGCTGAGC) was synthesized and cloned into pRNAe-egfpc1 with XhoI/NotI |
| pRNAe-c-myb | This work | Vector for RNAe targeting endogenous c-myb mRNA expression | Relative pairing sequence (CTATATATGCTGTGCCGGGGTCTTCGGGCCATGGCGCGGCGGGCGGCGGGGCTCCGCCGAGAGCCGCGGGGA) was synthesized and cloned into pRNAe-egfpc1 with XhoI/NotI |
| pRNAe-erbb2 | This work | Vector for RNAe targeting endogenous erbb2 mRNA expression | Relative pairing sequence (AGCCCCCAGCGGCACAAGGCCGCCAGCTCCATGGTGCTCACTGCGGCTCCGGCCCCATGGCTCCGGCTGGAC) was synthesized and cloned into pRNAe-egfpc1 with XhoI/NotI |
| pRNAe-bcl-xL | This work | Vector for RNAe targeting endogenous bcl-xl mRNA expression | Relative pairing sequence (TCAACCACCAGCTCCCGGTTGCTCTGAGACATTTTTATAATAGGGATGGGCTCAACCAGTCCATTGTCCAAA) was synthesized and cloned into pRNAe-egfpc1 with XhoI/NotI |
| pRNAe-bcl-2 | This work | Vector for RNAe targeting endogenous bcl-2 mRNA expression | Relative pairing sequence (TTATCGTACCCTGTTCTCCCAGCGTGCGCCATCCTTCCCAGAGGAAAAGCAACGGGGGCCAACGGCACCTCT) was synthesized and cloned into pRNAe-egfpc1 with XhoI/NotI |
| pRNAe-apc | This work | Vector for RNAe targeting endogenous apc mRNA expression | Relative pairing sequence (AGCTGCTCGTAGGGCGCCACGGAGCTCGCCATCTTCAGCTCCTGCAGCGTCCGCTTACGTGCAGCTGGGCTG) was synthesized and cloned into pRNAe-egfpc1 with XhoI/NotI |
| pRNAe-ctnnb1 | This work | Vector for RNAe targeting endogenous ctnnb1 mRNA expression | Relative pairing sequence (TCCAACTCCATCAAATCAGCTTGAGTAGCCATTGTCCACGCTGGATTTTCAAAACAGTTGTATGGTATACTT) was synthesized and cloned into pRNAe-egfpc1 with XhoI/NotI |
| pRNAe-bax | This work | Vector for RNAe targeting endogenous bax mRNA expression | Relative pairing sequence (CCGCCTCTGGGCTGCTCCCCGGACCCGTCCATCACCGCCGCTCCCGCCGCCGCCTCTCGCCGGGTCCGCGCG) was synthesized and cloned into pRNAe-egfpc1 with XhoI/NotI |
| pRNAe-c-myc | This work | Vector for RNAe targeting endogenous c-myc mRNA expression | Relative pairing sequence (TTCCTGTTGGTGAAGCTAACGTTGAGGGGCATCGTCGCGGGAGGCTGCTGGTTTTCCACTACCCGAAAAAAA) was synthesized and cloned into pRNAe-egfpc1 with XhoI/NotI |
| pRNAe-src | This work | Vector for RNAe targeting endogenous src mRNA expression | Relative pairing sequence (GCATCCTTGGGCTTGCTCTTGTTGCTACCCATGGTCCTGGTAGAAGGCAGGGGCTGTCCCAGAGGACCTGGG) was synthesized and cloned into pRNAe-egfpc1 with XhoI/NotI |
| pRNAe-p53 | This work | Vector for RNAe targeting endogenous p53 mRNA expression | Relative pairing sequence (TCGACGCTAGGATCTGACTGCGGCTCCTCCATGGCAGTGACCCGGAAGGCAGTCTGGCTGCCAATCCAGGGA) was synthesized and cloned into pRNAe-egfpc1 with XhoI/NotI |
| pRNAe-sp1 | This work | Vector for RNAe targeting endogenous sp1 mRNA expression | Relative pairing sequence (ATTTCATCCATGGAGTGATCTTGGTCGCTCATGGTGGCAGCTGAGGGACAAGCTCAAGGGGGTCCTGTCCGG) was synthesized and cloned into pRNAe-egfpc1 with XhoI/NotI |
| pRNAe-h-ras | This work | Vector for RNAe targeting endogenous h-ras mRNA expression | Relative pairing sequence (GCGCCCACCACCACCAGCTTATATTCCGTCATCGCTCCTCAGGGGCCTGCGGCCCGGGGTCCTCCTACAGGG) was synthesized and cloned into pRNAe-egfpc1 with XhoI/NotI |

**Supplementary Table 4** Primers and probes used in the article

| Name | Sequence (5’→3’) | Other |
| --- | --- | --- |
| GFP-f | CTACAACAGCCACAACGTCTATATCA |  |
| GFP-r | ATGTTGTGGCGGATCTTGAAG |  |
| GFP-probe | CGACAAGCAGAAGAACGGCATCAAGG | 5'FAM/3'Eclipse |
| GAPDH-f | GGAAGGTGAAGGTCGGAGTCA |  |
| GAPDH-r | TGGAAGATGGTGATGGGATTTC |  |
| GAPDH-probe | GATGACAAGCTTCCCGTTCTCAGCC | 5'FAM/3'Eclipse |
| upstream-f | ATCCAGCACAGTGGCGGC |  |
| upstream-r | GCTAACTAGAGAACCCACTGCTTACTG |  |
| upstream-probe | CGACTCACTATAGGGAGACCCAAGCTGG | 5'FAM/3'Eclipse |
| downstream-f | GGTACGCTACAGAGACTCGGGA |  |
| downstream-r | CGCATGCATCAACAGACCTCT |  |
| downstream-probe | ACTGTGAGTGGCTGACTGGCATGG | 5'FAM/3'Eclipse |
| 3'RACE-primer-1 | CAGTGCTAGAGGAGGTCAGAAGA |  |
| 3'RACE-primer-2 | TGAGCCATCTCTTTAGCTCCAGT |  |
