# Supplementary material for: RNAe: an effective method for targeted protein translation enhancement by artificial non-coding RNA with SINEB2 repeat
Source: Nucleic Acids Res. 2015 Feb 26;43(9):e58. doi: 10.1093/nar/gkv125 (PMC4482056; doi:10.1093/nar/gkv125)
Supplement: SUPPLEMENTARY DATA [file supp_gkv125_nar-02547-met-g-2014-File014.docx]

**Supplementary Reference**

1. Koshida, Y., Saegusa, M. and Okayasu, I. (1997) Apoptosis, cell proliferation and expression of Bcl-2 and Bax in gastric carcinomas: immunohistochemical and clinicopathological study. *Brit. J. Cancer*, **75**, 367.
2. Boise, L.H., González-García, M., Postema, C.E., Ding, L., Lindsten, T., Turka, L.A., Mao, X., Nuñez, G. and Thompson, C.B. (1993) bcl-x, a bcl-2-related gene that functions as a dominant regulator of apoptotic cell death. *Cell*, **74**, 597-608.
3. Pils, D., Bachmayr-Heyda, A., Auer, K., Svoboda, M., Auner, V., Hager, G., Obermayr, E., Reiner, A., Reinthaller, A. and Speiser, P., et al. (2014) Cyclin E1 (CCNE1) as independent positive prognostic factor in advanced stage serous ovarian cancer patients–A study of the OVCAD consortium. *Eur. J. Cancer*, **50**, 99-110.
4. Smeyne, R.J., Vendrell, M., Hayward, M., Baker, S.J., Miao, G.G., Schilling, K., Robertson, L.M., Curran, T. and Morgan, J.I. (1993) Continuous c-fos expression precedes programmed cell death in vivo. *Nature*, **363**, 166-169.23
5. 、Korinek, V., Barker, N., Morin, P.J., van Wichen, D., de Weger, R., Kinzler, K.W., Vogelstein, B. and Clevers, H. (1997) Constitutive transcriptional activation by a β-catenin-Tcf complex in APC−/− colon carcinoma. *Science*, **275**, 1784-1787.
6. Yamada, S., Shimada, M., Utsunomiya, T., Morine, Y., Imura, S., Ikemoto, T., Mori, H., Arakawa, Y., Kanamoto, M. and Iwahashi, S., et al. (2013) CXC receptor 4 and stromal cell–derived factor 1 in primary tumors and liver metastases of colorectal cancer. *J. Surg. Res.*, **187**, 107-112.
7. Khosravi, S., Wong, R.P., Ardekani, G.S., Zhang, G., Martinka, M., Ong, C.J. and Li, G. (2014) Role of EIF5A2, a downstream target of Akt, in promoting melanoma cell invasion. *Brit. J. Cancer*, **110**, 399-408.
8. Kallioniemi, O.P., Holli, K., Visakorpi, T., Koivula, T., Helin, H.H. and Isola, J.J. (1991) Association of C-erbB-2 protein over-expression with high rate of cell proliferation, increased risk of visceral metastasis and poor long-term survival in breast cancer. *Int. J. Cancer*, **49**, 650-655.
9. Sakai, E., Rikimaru, K., Ueda, M., Matsumoto, Y., Ishii, N., Enomoto, S., Yamamoto, H. and Tsuchida, N. (1992) The p53 tumor-suppressor gene and ras oncogene mutations in oral squamous-cell carcinoma. *Int. J. Cancer*, **52**, 867-872.
10. Barnes, J. A., Dix, D. J., Collins, B. W., Luft, C. and Allen, J. W. (2001) Expression of inducible Hsp70 enhances the proliferation of MCF-7 breast cancer cells and protects against the cytotoxic effects of hyperthermia. *Cell stress Chaperon*, **6**, 316.
11. Haigis, K.M., Kendall, K.R., Wang, Y., Cheung, A., Haigis, M.C., Glickman, J.N., Niwa-Kawakita, M., Sweet-Cordero, A., Sebolt-Leopold, J. and Shannon, K.M., et al. (2008) Differential effects of oncogenic K-Ras and N-Ras on proliferation, differentiation and tumor progression in the colon. *Nat. Genet.*, **40**, 600-608.
12. Tsai, S., Hollenbeck, S.T., Ryer, E.J., Edlin, R., Yamanouchi, D., Kundi, R., Wang, C., Liu, B. and Kent, K.C. (2009) TGF-β through Smad3 signaling stimulates vascular smooth muscle cell proliferation and neointimal formation. AM. J. Physiol-Heart C., 297, H540.
13. Lu, S. & Archer and M. C. (2010) Sp1 coordinately regulates de novo lipogenesis and proliferation in cancer cells. *Int. J. Cancer,* **126**, 416-425.
14. Irby, R., Mao, W., Coppola, D., Jove, R., Gamero, A., Cuthbertson, D., Fujita, D.J. and Yeatman, T.J. (1997) Overexpression of normal c-Src in poorly metastatic human colon cancer cells enhances primary tumor growth but not metastatic potential. *Cell Growth Differ.* , **8**, 1287-1295
15. Bai, L., Ni, H. M. & Chen, X., DiFrancesca, D. and Yin, X. M. (2005) Deletion of Bid impedes cell proliferation and hepatic carcinogenesis. *AM. J. Pathol*., **166**, 1523-1532.
16. Majello, B., Kenyon, L. C. and Dalla-Favera, R. (1986) Human c-myb protooncogene: nucleotide sequence of cDNA and organization of the genomic locus. *PNAS*, **83**, 9636-9640.
17. Tsai, K.Y., Hu, Y., Macleod, K.F., Crowley, D., Yamasaki, L. and Jacks, T. (1998) Mutation of E2f-1 Suppresses Apoptosis and Inappropriate S Phase Entry and Extends Survival of Rb-Deficient Mouse Embryos. *Mol. Cell*, **2**, 293-304.
18. Desai, S. S., Modali, S. D., Parekh, V. I., Kebebew, E. and Agarwal, S. K. (2014) GSK-3β Phosphorylates and Stabilizes HLXB9 in Insulinoma Cells to Form a Targetable Mechanism of Controlling Insulinoma Cell Proliferation. *J. Biol. Chem*. jbc-**M113**, 533-612.
19. Xiong, Y., Hannon, G.J., Zhang, H., Casso, D., Kobayashi, R. and Beach, D. (1993) p21 is a universal inhibitor of cyclin kinases. *Nature*, **366**, 701-704
20. Strasser, A., Harris, A.W., Jacks, T. and Cory, S. (1994) DNA damage can induce apoptosis in proliferating lymphoid cells via p53-independent mechanisms inhibitable by Bcl-2. *Cell*, **79**, 329-339.
21. Mahmoud, N.N., Boolbol, S.K., Bilinski, R.T., Martucci, C., Chadburn, A. and Bertagnolli, M.M. (1997) Apc gene mutation is associated with a dominant-negative effect upon intestinal cell migration. *Cancer Res.*, **57**, 5045-5050.
22. Wu, K.J., Grandori, C., Amacker, M., Simon-Vermot, N., Polack, A., Lingner, J. and Dalla-Favera, R. (1999) Direct activation of TERT transcription by c-MYC. *Nat. Genet*., **21**, 220-224.
23. Vafa, O., Wade, M., Kern, S., Beeche, M., Pandita, T.K., Hampton, G.M. and Wahl, G.M. (2002) c-Myc can induce DNA damage, increase reactive oxygen species, and mitigate p53 function: a mechanism for oncogene-induced genetic instability. *Mol. Cell*, **9**, 1031-1044.
24. Yao, Y., He, Y., Guan, Q. & Wu, Q. A tetracycline expression system in combination with Sox9 for cartilage tissue engineering. *Biomaterials,* **35**, 1898-1906 (2014).
25. Carrieri, C., Cimatti, L., Biagioli, M., Beugnet, A., Zucchelli, S., Fedele, S., Pesce, E., Ferrer, I., Collavin, L. and Santoro, C., et al. (2013) Long non-coding antisense RNA controls Uchl1 translation through an embedded SINEB2 repeat. *Nature*, **491**, 454-457.
26. Zhang, K., Zhao, W.J., Leng, X.Y., Wang, S., Yao, K. and Yan, Y. (2014) The importance of the last strand at the C-terminus inβB2-crystallin stability and assembly. *BBA-Mol. Basis Dis.*, **1842**, 44-55.
27. Yu, Y., Tong, P., Li, Y., Lu, Z. and Chen, Y. (2014) 10E8-like neutralizing antibodies against HIV-1 induced using a precisely designed conformational peptide as a vaccine prime. *Sci. China C. Life Sci.*, **57**, 117-127.
